# Engineering advances in polymer-modified bitumen for sustainable road construction: a review

**DOI:** 10.1039/d6ra06361f

**Published:** 2026-10-27

**Authors:** Yernar Kanzharkan, Yerbol Tileuberdi, Yerdos Ongarbayev, Yerzhan Imanbayev, Elmira Nurgaziyeva, Munziya Abutalip

**Affiliations:** a Institute of Combustion Problems Almaty Kazakhstan; b Faculty of Natural Science and Geography, Abay Kazakh National Pedagogical University Almaty Kazakhstan; c Faculty of Chemistry and Chemical Technology, Al-Farabi Kazakh National University Almaty Kazakhstan; d The Institute of New Materials and Energy Technologies, Nazarbayev University Astana Kazakhstan elmira.nurgaziyeva@nu.edu.kz; e Weill Cornell Medicine, Cornell University New York USA munziya.abutalip@nu.edu.kz

## Abstract

Road infrastructure is under increasing pressure from heavier traffic, wider temperature fluctuations, accelerated aging, and stricter sustainability targets. Conventional bitumen, although widely used as a binder in asphalt pavements, often shows inadequate resistance to rutting at high temperatures, fatigue damage under repeated loading, and thermal cracking at low temperatures. Polymer modification has therefore become one of the most effective strategies for improving binder performance and extending pavement service life. Recent studies show that elastomers, plastomers, recycled polymers, and crumb rubber can substantially enhance the viscoelastic balance, durability, and temperature susceptibility of asphalt binders. At the same time, the field is evolving beyond conventional modification toward multifunctional systems integrating waste-derived materials, nanomaterials, self-healing concepts, and smart sensing technologies. Rather than treating polymer modifiers as isolated material classes, this review establishes a structure–interaction–performance framework linking modifier chemistry and processing with polymer–bitumen interactions, morphology evolution, storage stability, binder rheology, and asphalt-mixture and pavement-level performance. The review therefore considers polymer modification across multiple scales, from binder-level physicochemical and rheological behavior to relevant mixture-level mechanical performance and pavement implications. The framework further integrates emerging sustainable and multifunctional strategies, including waste-derived modifiers, polymer-assisted oxidative upgrading, nanomaterials, self-healing systems, and smart pavement technologies.

## Abstract 2

Road infrastructure is under increasing pressure from heavier traffic, wider temperature fluctuations, accelerated aging, and stricter sustainability targets.

## Graphical abstract

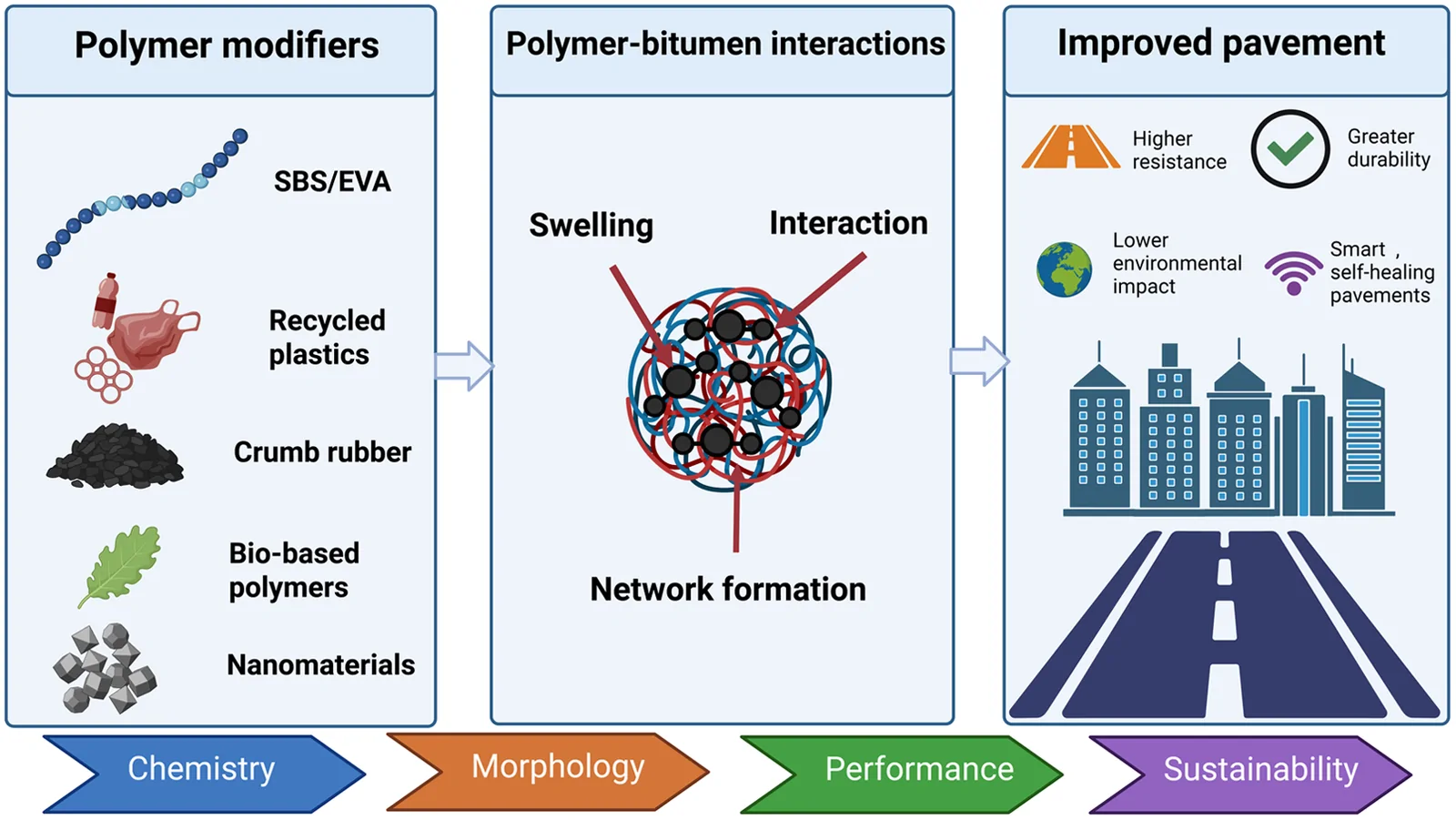

## 1. Introduction

Transportation infrastructure plays a fundamental role in economic growth, social connectivity, and sustainable development.^1,2^ Among various transportation systems, road networks constitute the backbone of modern mobility, carrying the majority of passenger and freight traffic worldwide.^1^ The continuous increase in traffic volume, axle loads, and vehicle speeds, coupled with climate-change-related temperature variations, has intensified the demand for high-performance pavement materials with long-term durability under severe service conditions.^3,4^ Consequently, improving the mechanical performance, sustainability, and service life of asphalt pavements has become a major priority for pavement researchers and transportation agencies.^5,6^

In this review, the terms bitumen and asphalt binder refer to the petroleum-derived binding material used to coat and bind mineral aggregates, whereas asphalt mixture or asphalt concrete refers to the composite pavement material consisting primarily of mineral aggregates and bituminous binder, with or without additional fillers and additives.

Bitumen, a viscoelastic petroleum-derived binder, is widely used in flexible pavements because of its adhesive properties, water resistance, and processability.^7,8^ However, conventional bitumen exhibits several limitations, including rutting at high temperatures, thermal cracking at low temperatures, fatigue damage under repeated traffic loading, and oxidative aging during service.^8–11^ These problems often lead to increased maintenance needs, reduced pavement lifespan, and significant economic and environmental impacts.^5,12^ Therefore, more durable and environmentally responsible pavement technologies are urgently required.^12,13^

A broad range of additives and modification strategies has been developed to tailor the technological and performance characteristics of road bitumen. These include polymeric modifiers, plasticizers and rejuvenating agents, adhesion promoters and antistripping additives, antioxidants and anti-aging agents, mineral and nanoscale additives, and renewable or waste-derived materials that can partially substitute or modify petroleum bitumen. Polymeric modifiers themselves comprise several major classes, including thermoplastics such as polyethylene (PE), polypropylene (PP), and ethylene-vinyl acetate (EVA); elastomers and thermoplastic elastomers such as styrene–butadiene rubber (SBR) and styrene–butadiene–styrene (SBS); and reactive or functionalized polymers.^14^ Plasticizers and rejuvenators are primarily used to improve flexibility and restore the properties of aged binders, whereas antioxidants are introduced to retard oxidative hardening during production and service.^15^ Adhesion promoters act mainly at the bitumen-aggregate interface to improve coating and resistance to moisture-induced debonding.^16^ These additive classes therefore address different limitations of conventional bitumen and should not be regarded as mechanistically equivalent.

Polymer modification has emerged as one of the most effective approaches to improve the performance of bituminous binders.^17–19^ The incorporation of polymers can enhance elasticity, stiffness, temperature susceptibility, rutting resistance, fatigue resistance, and overall pavement durability.^17–21^ Commonly used modifiers such as styrene–butadiene–styrene (SBS), styrene–butadiene rubber (SBR), ethylene-vinyl acetate (EVA), polyethylene (PE), polypropylene (PP), and crumb rubber have demonstrated strong potential for improving asphalt performance under different climatic and traffic conditions.^18,20–24^

Recent advances in materials science, nanotechnology, and sustainable engineering have further expanded the scope of polymer-modified bitumen (PMB). Nanoclays, carbon-based nanomaterials, functionalized polymers, graphene derivatives, bio-based modifiers, recycled plastics, and hybrid composite systems have been investigated to improve compatibility, storage stability, aging resistance, and long-term pavement performance.^25–30^ At the same time, circular economy principles have encouraged the use of waste polymers, crumb rubber, and bio-derived materials as sustainable alternatives to conventional modifiers.^23,24,29,30^

In parallel, emerging technologies such as warm-mix asphalt, self-healing asphalt materials, intelligent pavement systems, machine-learning-assisted mixture design, and digital pavement monitoring are reshaping modern road construction.^31–34^ The integration of advanced polymer-modified binders with these technologies offers new opportunities to develop resilient, smart, and sustainable transportation infrastructure (Fig. 1).

**Fig. 1:**
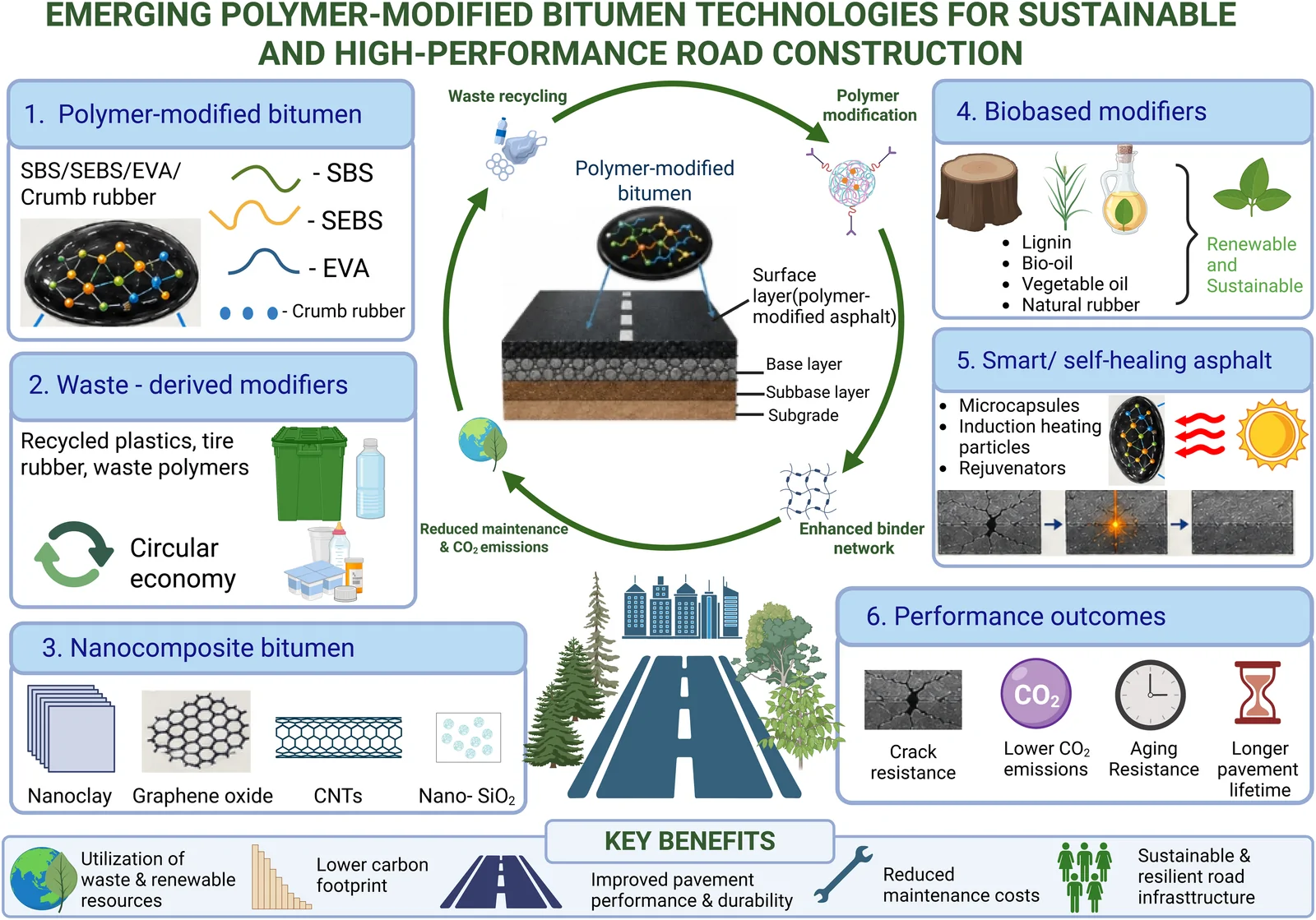
Overview of emerging polymer-modified bitumen technologies for sustainable and high-performance road construction.

A schematic overview of the evolution of polymer-modified bitumen technologies for future road construction is presented in Fig. 2.

**Fig. 2:**
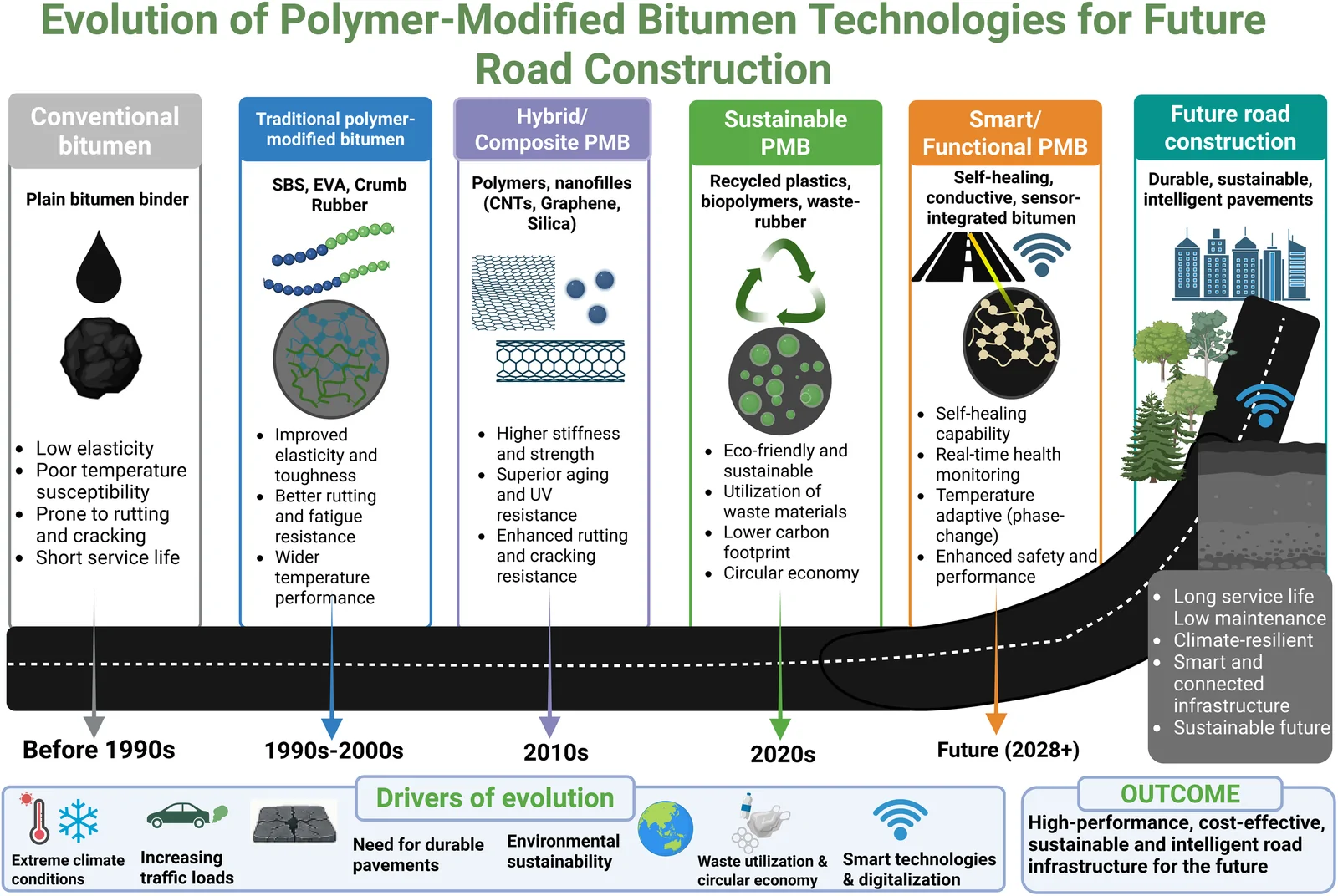
Evolution of polymer-modified bitumen technologies from conventional binders to sustainable and smart pavement systems.

Despite these advances, important challenges remain in polymer–bitumen compatibility, phase separation, aging resistance, economic feasibility, large-scale implementation, and environmental assessment.^17–19,27^ In particular, differences in polymer polarity, density, and interfacial tension can promote instability, while aging may progressively deteriorate polymer networks and rheological performance.^35^ Alnadish *et al.*^35^ further emphasized that improving PMB durability requires not only appropriate polymer selection but also optimization of blending conditions, including mixing sequence, shear rate, viscosity, and mixing time, together with compatibilizers, cross-linking agents, and other stabilization strategies. These limitations indicate that PMB performance cannot be understood solely from polymer type or reported mechanical improvement but must also be considered in relation to polymer–bitumen interactions, processing conditions, morphology evolution, storage stability, and long-term durability.

Existing reviews have provided important insights into fundamental bitumen science, conventional polymer modification, polymer–bitumen interactions, individual modifier classes, recycled polymers, nanomaterials, self-healing asphalt, and smart pavement technologies. Table 1 summarizes representative reviews and their principal areas of emphasis, providing the basis for identifying the remaining gap addressed in the present work.

**Table 1:** Major review papers on polymer-modified bitumen and their thematic focus

Author	Year	Scope	Main focus	Relevance to future roads	Reference
Lesueur	2009	Fundamental bitumen science	Colloidal structure of bitumen, rheology, and mechanisms of bitumen modification	Provides the theoretical basis for designing advanced binders for durable pavements	7
Yildirim	2007	General review of polymer-modified asphalt binders	Performance benefits of polymer-modified binders in asphalt pavements	Establishes PMB as a key material for high-performance road construction	17
Zhu *et al.*	2014	Broad review of polymer modification of bitumen	Advances, challenges, compatibility, storage stability, and modifier selection	Highlights technical barriers and opportunities for next-generation PMB systems	18
Emtiaz *et al.*	2023	Comprehensive review of polymer-modified asphalt binder	Binder types, preparation methods, performance, and characterization	Supports updated understanding of PMB technologies for modern pavement design	36
Yang *et al.*	2024	Mechanistic review of polymer-modified asphalt binders	Modification mechanisms, polymer–bitumen interactions, and mechanical properties	Directly links PMB mechanisms with future high-performance road materials	37
Pipintakos *et al.*	2024	Future-oriented review of bitumen engineering	Chemistry, structure, rheology, and engineering of bitumen for advanced pavements	Strongly aligned with future road construction and advanced binder design	38
Duarte and Faxina	2021	Polymeric waste in asphalt mixtures	Wet and dry processing of waste-polymer-modified asphalt mixtures	Demonstrates the role of recycled polymers in sustainable road infrastructure	39
Guerrero-Bustamante *et al.*	2024	Crumb rubber in road pavements	Use of crumb rubber in binders and mixtures, performance, and implementation	Relevant for circular economy approaches and durable rubberized roads	40
Afshin and Behnood	2025	Nanomaterials in asphalt pavements	Types of nanomaterials, mechanisms, and pavement performance enhancement	Supports the development of multifunctional and high-performance future pavements	41
He *et al.*	2024	Self-healing asphalt technologies	Self-healing mechanisms, repair strategies, and material design	Highly relevant for long-life, low-maintenance future road systems	42
Xu *et al.*	2021	Waste plastics as asphalt modifiers	Plastic waste utilization, binder modification, and sustainability aspects	Connects waste valorization with sustainable pavement engineering	43
Abdy *et al.*	2022	Pyrolysis-derived polyolefin waste for asphalt	Plastic waste pyrolysis and its applications in asphalt road construction	Relevant for sustainable material recovery and low-carbon future roads	44
Wan *et al.*	2023	Extrinsic self-healing asphalt materials	Self-healing strategies based on external agents and functional additives	Contributes to resilient pavements with extended service life	45
Di Graziano *et al.*	2020	Smart sensor networks in asphalt pavements	Structural health monitoring and smart pavement systems	Relevant for digitalized and intelligent transport infrastructure	46
Hossein *et al.*	2024	Smart embedded pavement technologies	Advanced embedded materials and technologies for pavement management	Supports future roads with integrated sensing and monitoring capabilities	47
Da *et al.*	2025	Automated asphalt pavement inspection technologies	Smart materials, automated inspection, and infrastructure digitalization	Directly relevant to intelligent and data-driven future road construction	48

Accordingly, the scope of this review extends from binder-level physicochemical, morphological, and rheological behavior to relevant asphalt-mixture and pavement-level performance, while recognizing that binder-scale improvements do not necessarily translate directly into equivalent mixture- or field-scale performance.

Although recent reviews have addressed polymer-modification mechanisms, individual modifier classes, waste-derived materials, nanomaterials, self-healing asphalt, and smart pavement technologies, these topics have largely been discussed as separate aspects of asphalt modification. Accordingly, the objective of the present review is to integrate these previously separated research directions within a unified structure–interaction–performance framework that traces how modifier chemistry and processing govern polymer swelling, interfacial interactions, phase morphology, and network formation, and how these features subsequently determine rheological behavior, storage stability, durability, and pavement-level performance. This framework also extends beyond conventional physical polymer modification by considering emerging pathways in which waste polymers may participate in binder restructuring during oxidative upgrading, together with hybrid, sustainable, self-healing, and smart material strategies. Thus, rather than introducing another classification of polymer modifiers, this review provides a mechanistic basis for understanding the transition from modifier selection and processing to stable microstructure, engineering performance, and ultimately sustainable and multifunctional pavement systems.

## 2. Fundamentals of bitumen and performance limitations of conventional binders

Bitumen is a highly complex thermoviscoelastic material derived from crude oil refining and commonly interpreted through the interactions among saturates, aromatics, resins, and asphaltenes.^49^ Its chemistry and colloidal organization largely determine its rheological response, stiffness evolution, temperature susceptibility, and long-term mechanical behavior. Lesueur showed that the mechanical performance of bitumen cannot be separated from its internal colloidal structure, while more recent work has reinforced the importance of linking chemistry, structure, and rheology in binder design for future pavements.^38,49^

In practical pavement systems, bitumen serves as the binding phase that coats mineral aggregates, provides cohesion, and contributes to load distribution and flexibility. However, conventional binders must operate under highly variable traffic and environmental conditions, which creates competing performance demands. At elevated temperatures, insufficient stiffness and flow resistance can lead to rutting and permanent deformation. Under repeated traffic loading at intermediate temperatures, pavements become vulnerable to fatigue cracking. At low temperatures, the binder may lose relaxation capacity and become brittle, increasing the likelihood of thermal cracking. These temperature-dependent failures make the performance limitations of conventional binders one of the central challenges in modern asphalt engineering.^49,50^

Another major limitation of conventional binders is aging. During asphalt production, short-term aging occurs because of heating, volatilization of lighter components, and oxidation during mixing and compaction. During field service, long-term aging continues under oxygen exposure, thermal cycling, solar radiation, and environmental action. These processes progressively alter binder chemistry, increase stiffness, reduce ductility, and weaken the balance between strength and flexibility. As a result, aged binders often show greater susceptibility to cracking and reduced long-term durability.^49^

Because of these limitations, conventional bitumen often fails to satisfy the increasingly demanding requirements of modern road infrastructure, especially under heavy axle loads, climatic extremes, and longer design-life expectations. This performance gap has been one of the main drivers behind binder modification strategies, particularly polymer modification. Reviews by Polacco and Yang *et al.* indicate that modification approaches are intended to improve the viscoelastic balance of binders, enhance rutting and fatigue resistance, and extend service performance beyond what is typically achievable with base bitumen alone.^37,50^

In addition, the behavior of conventional and modified binders is strongly influenced not only by material composition but also by preparation and testing methodology. Recent reviews emphasize that reliable interpretation of binder performance requires attention to blending conditions, temperature history, shear intensity, and characterization strategy.^51^ For this reason, understanding the limitations of conventional binders should be treated as the starting point for a more advanced discussion of polymer modification, morphology evolution, rheological performance, and future-oriented binder design (Fig. 3).

**Fig. 3:**
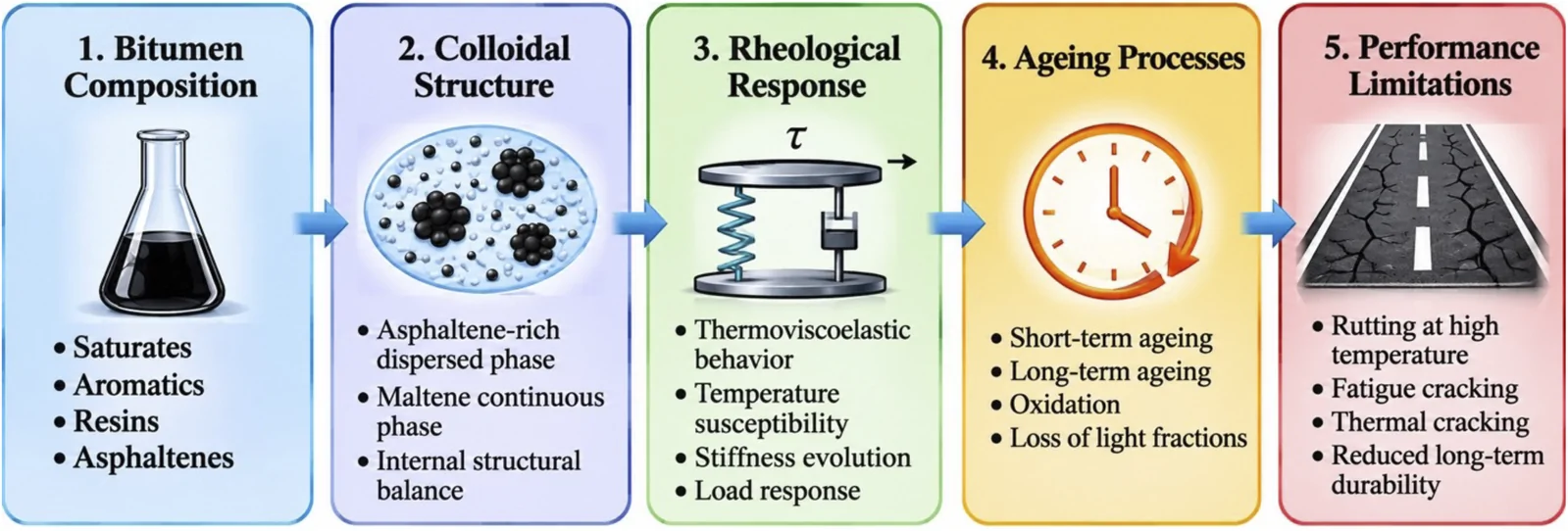
Schematic overview of the composition, colloidal structure, ageing behavior, and performance limitations of conventional bitumen in pavement applications.

## 3. Polymer modification of bitumen: classification of modifiers

Polymer modification of bitumen is widely used to improve the service performance of asphalt binders by tailoring stiffness, elasticity, storage stability, and durability. Reviews over the last two decades show that modifier selection is one of the most important variables in PMB design, because different polymer classes produce different microstructures and performance balances in the binder.^50,52^ In general, polymer modifiers can be grouped into elastomers, plastomers, recycled polymers, reactive or functionalized polymers, and hybrid modifier systems, each of which contributes differently to pavement performance and long-term binder behavior.^37^ The main classes of polymer modifiers used in bitumen modification are summarized in Fig. 4.

**Fig. 4:**
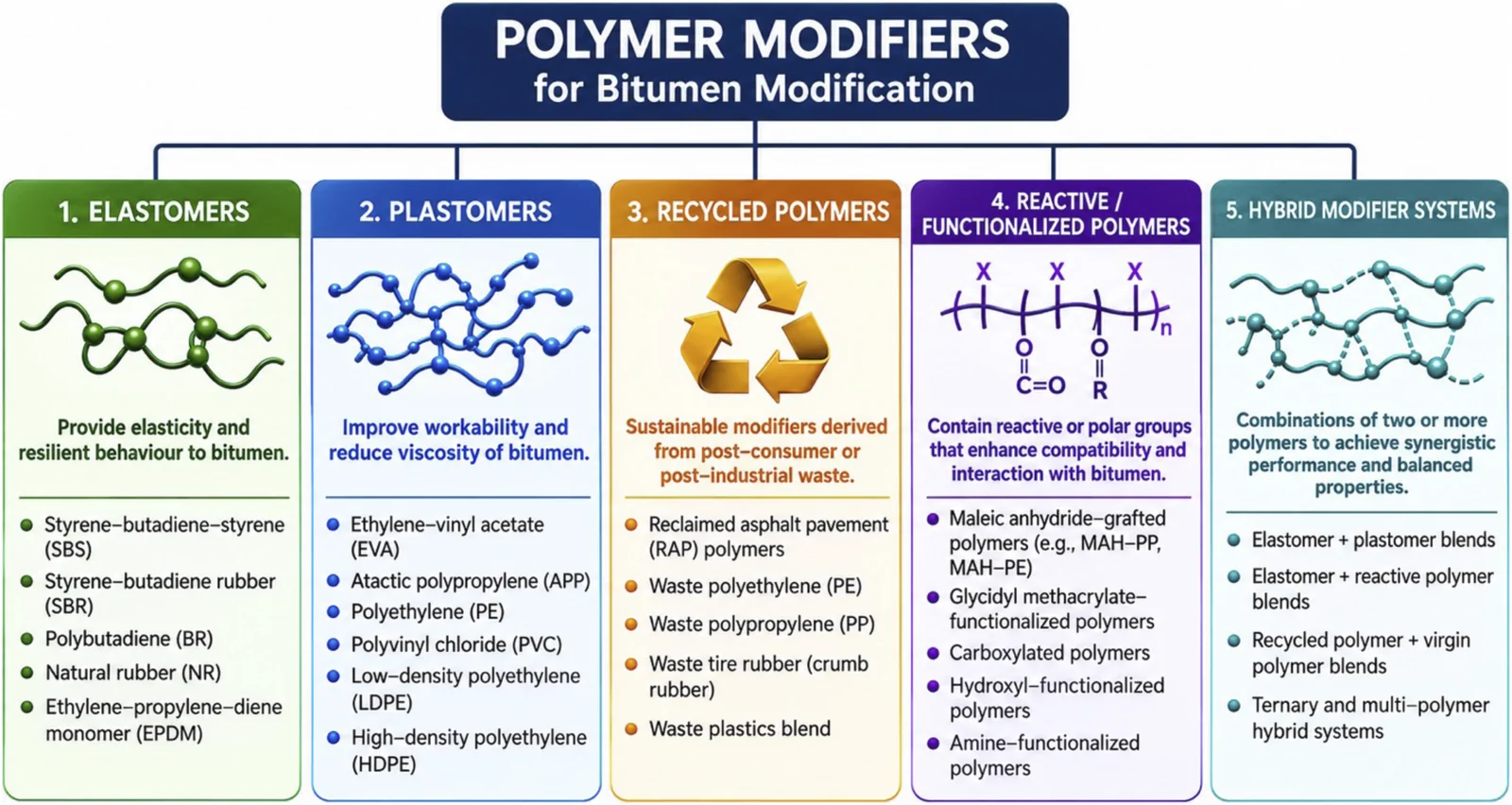
Classification of polymer modifiers used in bitumen modification.

Although different polymer modifiers improve bitumen performance through distinct mechanisms, their overall effectiveness depends on the interaction between polymer chemistry, thermal characteristics, and rheological behavior. Ishaq and Giustozzi^51^ demonstrated that the performance of polymer-modified binders is governed not only by polymer type but also by their thermo-chemical characteristics. By combining FTIR, DSC, TGA, MSCR, LAS, and Glover–Rowe analyses, they showed that elastomers provide superior fatigue resistance and low-temperature flexibility owing to their low glass transition temperature, whereas plastomers improve stiffness, rutting resistance, and thermal stability because of their higher crystallinity. Furthermore, incorporating microfibres enhanced cohesive strength and produced a more balanced rheological response over a broad temperature range, highlighting the importance of selecting modifiers according to the target pavement conditions rather than relying on a single polymer type.

### 3.1 Elastomers

Elastomeric modifiers are primarily used to improve elasticity, recoverable deformation, and fatigue resistance in asphalt binders. The most common elastomers include styrene–butadiene–styrene (SBS), styrene–butadiene rubber (SBR), and related thermoplastic elastomers. These polymers are especially valued because they can form an elastic network within the bitumen phase, which enhances the viscoelastic balance of the binder and improves resistance to rutting and fatigue cracking under traffic loading.^50,52^ Among them, SBS remains the most extensively used modifier in practice because of its broad performance benefits and commercial maturity.^37^

Recent research has focused not only on improving the performance of SBS-modified bitumen but also on simplifying its production process. Akimov *et al.*^53^ developed an innovative polymer–bitumen concentrate (PBC) comprising SBS, a plasticizer, and surfactants, enabling efficient polymer incorporation into bitumen without the need for high-shear colloid milling. Compared with conventional SBS modification, this approach reduced processing viscosity and energy consumption while improving polymer dispersion and mitigating thermo-oxidative aging during production. The synergistic action of the plasticizer and surfactants enhanced polymer compatibility with bitumen, resulting in higher elasticity, improved adhesion, increased cohesive strength, and an expanded plasticity temperature range. At an optimum PBC content of 8 wt%, the modified binder achieved a PG 64–28 performance grade with rheological properties superior to those of conventionally produced SBS-modified bitumen. These findings demonstrate that advances in processing technology can be as important as polymer formulation itself, offering a practical route toward more energy-efficient and economically viable production of SBS-modified binders.

Laukkanen *et al.*^54^ provided important insights into the relationship between the microstructure and low-temperature rheology of SBS-modified bitumen. Fluorescence microscopy revealed that increasing the SBS concentration from 3 to 5 wt% induces phase inversion, forming a continuous SBS-rich network that significantly changes the viscoelastic response of the binder. This interconnected polymer network reduces temperature susceptibility, decreases creep stiffness, and enhances resistance to thermal cracking. However, excessive SBS contents also introduce thermorheological complexity because the polymer-rich and bitumen-rich phases exhibit different temperature-dependent relaxation mechanisms. The study further demonstrated that poor compatibility between SBS and bitumen promotes macro-phase separation, leading to deviations in rheological performance, whereas chemical crosslinking effectively suppresses phase separation and improves storage stability.

In addition to conventional SBS modification, recent experimental studies demonstrate the effectiveness of commercial polymer latices as elastomeric bitumen modifiers. Onyshchenko *et al.* investigated the cationic styrene–butadiene latex Butonal NX4190 at concentrations of 2–6 wt% and identified 2–4 wt% as the optimal range, with a preparation time of 3–4 h. At these dosages, the latex improved the physical and mechanical properties of the binder and produced substantial increases in asphalt-concrete strength.^55^ Depending on mixture type, compressive strength at 293 and 323 K increased by approximately 10–101%, while water-resistance coefficients approached 1.0 in some formulations. These results provide a quantitative example of how elastomeric latex modification can improve both binder- and mixture-level properties; however, the identified optimum remains dependent on latex dosage, processing conditions, and asphalt-mixture composition.

These findings highlight that the performance of SBS-modified bitumen depends not only on polymer dosage but also on the development of a stable and well-dispersed polymer network, emphasizing the critical role of polymer–bitumen compatibility in designing durable polymer-modified binders.

### 3.2 Plastomers

Plastomeric modifiers are generally selected to increase stiffness, rutting resistance, and high-temperature deformation resistance. Common representatives include polyethylene (PE), polypropylene (PP), ethylene-vinyl acetate (EVA), and ethylene-butyl acrylate (EBA). Unlike elastomers, which mainly enhance recoverable elasticity, plastomers tend to make the binder more rigid and less prone to permanent flow under heavy loads and warm climates.^52^ However, the literature also indicates that plastomer-rich systems can be more vulnerable to compatibility problems and may reduce low-temperature flexibility if the formulation is not properly balanced.^56^

Polyethylene (PE) is one of the most widely investigated plastomeric modifiers because it improves rutting resistance and stiffness while providing an environmentally beneficial route for recycling waste plastics. However, its practical application is often restricted by poor compatibility with bitumen, resulting in phase separation and inadequate storage stability. To overcome these limitations, Padhan and Sreeram^57^ demonstrated that the combined use of *trans*-polyoctenamer (TPOR) and sulfur-based cross-linking agents significantly enhanced the storage stability and rheological performance of PE-modified binders. Cross-linking promoted the formation of a stable three-dimensional polymer network, resulting in higher elastic recovery, improved rutting resistance, and a more homogeneous polymer distribution, as confirmed by fluorescence microscopy. These findings indicate that reactive modification is an effective strategy for improving the compatibility of recycled PE with bitumen and reducing phase separation while maintaining satisfactory workability. Despite these improvements, the long-term durability, oxidative aging resistance, and economic feasibility of reactive PE modification require further investigation before widespread field implementation.

Bulatović *et al.*^58^ systematically compared the effects of linear SBS (SBS-L), radial SBS (SBS-R), EVA, and reactive Elvaloy terpolymers on bitumen performance, demonstrating that polymer architecture plays a critical role in determining rheological behavior. All modifiers increased stiffness, softening point, and resistance to permanent deformation; however, radial SBS exhibited the greatest improvement in elasticity, complex modulus, and rutting resistance due to the formation of a more effective three-dimensional physical network within the bitumen matrix. EVA primarily enhanced stiffness and high-temperature performance but reduced low-temperature flexibility because of its semicrystalline nature. In contrast, reactive Elvaloy modifiers improved compatibility and storage stability through chemical bonding with asphaltenes, although excessive reactive group content promoted gel formation. These findings highlight that the balance between physical network formation (SBS), crystalline reinforcement (EVA), and chemical crosslinking (Elvaloy) governs the final performance of polymer-modified bitumen, emphasizing the importance of selecting both polymer type and concentration according to the targeted pavement service conditions.

### 3.3 Recycled polymers

#### 3.3.1 Recycled thermoplastic polymer modifiers

Recycled polymers have gained increasing attention because they combine performance enhancement with waste valorization and circular-economy benefits. Waste polyethylene, polypropylene, PET, mixed plastic waste, and other discarded polymer streams have been incorporated into asphalt binders and mixtures through both wet and dry processing routes. Reviews on waste-polymer asphalt systems show that recycled polymers can improve stiffness, rutting resistance, and in some cases mixture durability, although results depend strongly on polymer type, dosage, pretreatment, and blending route.^39,43^ Their main challenge remains variability in waste composition and compatibility with the base binder, which may affect reproducibility and long-term field stability.^39^

An emerging strategy for producing sustainable polymer-modified bitumen involves incorporating polymer waste directly during the oxidation of heavy oil residues rather than modifying the finished binder after production. This approach simultaneously upgrades refinery residues and recycles post-consumer plastics, offering a circular-economy alternative to conventional wet- and dry-process polymer modification. Unlike traditional PMB technologies, where polymers primarily improve binder properties through swelling and the formation of a continuous viscoelastic network, oxidative upgrading integrates polymer fragments into the evolving bitumen structure during oxidation, thereby modifying both the chemical composition and colloidal architecture of the binder.

Tileuberdi *et al.*^75^ demonstrated that low-density polyethylene (LDPE) waste can effectively participate in the oxidation of heavy oil residues under controlled air-blowing conditions. The chemical characterization provides valuable insight into the modification mechanism. FTIR analysis revealed increased intensities of carbonyl (≈1700 cm^−1^) and sulfoxide (≈1030 cm^−1^) absorption bands together with preserved aliphatic CH_2_ vibrations originating from polyethylene. These observations indicate that the polymer does not simply remain as an inert dispersed phase but becomes partially incorporated into the oxidized bitumen matrix while promoting the formation of oxygen-containing functional groups. Complementary ^1^H and ^13^C NMR analyses confirmed increased aromaticity, the appearance of carbonyl carbons (170–200 ppm), and detectable polyethylene-derived signals, suggesting that oxidative polymerization, aromatization, and partial integration of polyethylene fragments occur simultaneously during oxidation. Such molecular transformations promote the formation of a more stable and highly structured binder network than that obtained through oxidation alone.

The rheological results further support these structural changes. The binder produced using 2 wt% polyethylene waste at 270 °C for 4 h exhibited higher complex shear modulus (|G*|), lower temperature sensitivity, and improved structural stability over a broad frequency range compared with the binder produced under lower oxidation severity. The master curves indicate enhanced resistance to shear deformation under service conditions, implying superior rutting resistance and improved performance at elevated pavement temperatures. In addition, thermal analysis showed that the modified binders remained stable up to approximately 350 °C before significant decomposition occurred, confirming that polymer incorporation did not adversely affect thermal stability.

From the perspective of polymer-modified bitumen technology, this work highlights a fundamentally different modification pathway compared with conventional SBS-, EVA-, or crumb rubber-modified binders. In conventional PMB systems, polymer modification relies primarily on the diffusion of maltenes into the polymer phase, polymer swelling, and subsequent development of a continuous elastic network that governs the rheological response. In contrast, polymer-assisted oxidative upgrading modifies the binder at the molecular level through concurrent oxidation, aromatization, condensation, and partial incorporation of polymer fragments into the bitumen matrix. Consequently, the improved properties originate from chemical restructuring of the binder rather than solely from physical reinforcement by a swollen polymer phase. This distinction broadens the conceptual framework of polymer modification and demonstrates that polymer wastes may function not only as reinforcing additives but also as reactive components participating in binder synthesis.

Despite these promising findings, several limitations remain. The study focused exclusively on polyethylene waste and evaluated conventional physical, thermal, spectroscopic, and linear rheological properties, whereas long-term storage stability, fatigue resistance, low-temperature cracking behavior, moisture susceptibility, and field performance were not investigated. Furthermore, although FTIR and NMR results suggest partial incorporation of polyethylene fragments into the oxidized matrix, the exact reaction pathways and the extent of chemical bonding require further elucidation using advanced molecular characterization techniques. Future research should also investigate other waste polymers, including polypropylene, polyethylene terephthalate, multilayer plastic waste, and mixed municipal plastic streams, as well as hybrid systems combining recycled polymers with nanomaterials or conventional elastomers to achieve simultaneous improvements in high-temperature stability, elasticity, and long-term durability. Such developments could establish oxidative polymer-assisted upgrading as a promising next-generation technology for producing sustainable polymer-modified bitumen while simultaneously addressing plastic waste management challenges.

Recent studies have demonstrated that the effectiveness of cellulose as a bitumen modifier depends strongly on its chemical purification. Caputo *et al.*^59^ reported that acid- and bleaching-treated waste cellulose significantly improved the thermomechanical properties of bitumen by enhancing the dispersion of asphaltene-resin aggregates without markedly increasing binder stiffness. Density functional theory (DFT) analysis further confirmed that purified cellulose forms stable supramolecular complexes with asphaltene-resin clusters through weak non-covalent interactions, thereby reducing asphaltene aggregation and improving the stability of the colloidal network. However, untreated cellulose or excessive cellulose loading deteriorated binder performance, emphasizing that cellulose purification and dosage optimization are essential for successful bio-based bitumen modification. Ongarbayev *et al.*^60^ proposed new bitumen production and modification method in which crumb rubber (CR) is introduced both before and after vacuum residue oxidation, integrating bitumen production and modification into a single technological route.

#### 3.3.2 Recycled cross-linked elastomeric polymer modifier – crumb rubber

Crumb rubber (CR), produced from end-of-life tires, is one of the most extensively studied recycled cross-linked elastomeric modifiers for asphalt binders. Unlike thermoplastic polymers, crumb rubber consists of a vulcanized three-dimensional network that does not melt during asphalt mixing. Instead, CR particles absorb the light aromatic fractions of bitumen, swell, and form a physically reinforced network that enhances the viscoelastic properties of the binder. This swelling process improves elasticity, increases rutting resistance at high temperatures, and enhances fatigue and crack resistance under repeated traffic loading. Furthermore, the utilization of waste tires contributes to circular economy principles by reducing landfill disposal and promoting sustainable pavement construction. However, crumb-rubber-modified binders still face several challenges, including storage instability, high processing temperatures, increased viscosity, and variability associated with rubber particle size and source. Recent developments, such as terminal blending, microwave activation, devulcanization, and hybrid modification with nanomaterials or bio-based additives, have significantly improved compatibility and long-term performance, making crumb rubber an important component of next-generation sustainable asphalt technologies.^61^

### 3.4 Reactive/functionalized polymers

Reactive and functionalized polymers are used when improved interfacial interaction, phase stability, and chemical bonding are required. These modifiers are designed to interact more actively with the bitumen phase than conventional inert polymers, often resulting in better compatibility and reduced phase separation. Zhu *et al.* identify reactive polymers and functionalization strategies as important directions for overcoming the stability limitations of conventional PMB systems.^18^ A well-known example is reactive terpolymer technology such as Elvaloy® RET, which has been shown to improve rutting resistance and other performance properties, although dosage and processing conditions must be carefully controlled to avoid gel formation and instability.^50,62^

Wang *et al.*^63^ proposed a rational design strategy for multi-component waterborne polymer modifiers by combining conventional elastomeric and plastomeric polymers (SBS, SBR, and EVA) with reactive resin systems, including waterborne epoxy resin (WER), polyurethane (WPU), and acrylate (WA). Unlike conventional empirical approaches, the optimized ternary systems (WER/WPU/WA, WER/WPU/SBR-I, and WER/WA/SBR-I) exhibited superior tensile strength, bonding performance, flexibility, and elasticity due to the synergistic interaction between physical polymer networks and chemical cross-linking. The study further demonstrated that WPU chemically reacted with both WER and WA, while other polymer combinations mainly formed physical cross-linked structures, highlighting the importance of tailoring polymer interactions rather than relying solely on single modifiers. These findings suggest that hybrid waterborne polymer systems offer a promising route for developing high-performance, environmentally friendly emulsified asphalt with balanced mechanical properties and durability.

### 3.5 Hybrid modifiers

Hybrid modifier systems combine two or more modifier classes to achieve multifunctional performance that may not be attainable with a single additive alone. These systems may include polymer–rubber blends, polymer-nanomaterial systems, or multicomponent formulations containing polymers together with stabilizers or reactive additives. Recent reviews show that nanomaterial-assisted polymer modification, particularly in SBS-based binders, can improve viscosity, rutting resistance, fatigue resistance, aging resistance, and moisture-damage performance when dispersion is properly controlled.^64,65^ Hybrid systems are therefore increasingly viewed as promising candidates for future road construction, although they also introduce added complexity in formulation design, dispersion control, and cost-effectiveness.^64^ A comparative summary of the main polymer modifier classes, their functions, advantages, and limitations is presented in Table 2.

**Table 2:** Comparison of major bitumen modifiers: typical dosage ranges and representative performance effects

Modifier class	Typical examples	Typical dosage (wt% of binder)	Representative quantitative effects	Representative literature
Elastomers	SBS, SBR, SIS, SEBS	3–7%	Generally increases softening point and elastic recovery and decreases penetration. For BIT 70/100 a penetration of 71.1 dmm and softening point of 46.7 °C; with 2–7% linear SBS, penetration falls to 57–41 dmm, softening point rises to 53–90 °C, and elastic recovery increases from 47% at 2% SBS to 96.5% at 7% SBS	Bulatović *et al.*;^58^ other SBS studies cited in the manuscript
Elastomeric latex	Butonal NX4190 cationic styrene–butadiene latex	2–6%; optimum 2–4%	At the reported optimum dosage, asphalt-concrete compressive strength increased by **∼10–101%**, depending on mixture type and temperature; water-resistance coefficients approached **1.0** in some formulations	55
Plastomers – PE/PP	LDPE, HDPE, LLDPE, PP	3–5%	Representative literature values: PE can decrease penetration by ∼33–59% and increase softening point by ∼11–92%; PP can decrease penetration by ∼23–82% and increase softening point by ∼30–44%	72 and 73
Plastomers – EVA	EVA, EBA	1–10%; ∼5% commonly used	At 5% EVA, representative studies report penetration decreases of ∼33–51% and softening-point increases of ∼21.6–53%	72 and 73
Recycled thermoplastics	Waste PE, waste PP, waste PET, mixed plastics	∼3–5% for PE/PP; PET and mixed waste are formulation-dependent	Generally decreases penetration and increases softening point/stiffness; the magnitude is strongly dependent on waste composition, pretreatment, and processing route. For 3% PE, penetration reductions of 7–42% and softening-point increases of 2–50% were reported; at 5% PE, penetration reductions reached 22–63%, with softening-point increases of approximately 14–91.5%	72 and 73
Crumb rubber	Waste-tire rubber, GTR	12–20% commonly reported	Representative system (0 → 21% CR): penetration 48 → 27 dmm, softening point 50 → 72 °C, and resilience ∼0 → 47%	60, 61 and 74
Reactive/functionalized polymers	Elvaloy RET, GMA-functionalized terpolymers	0.5–1.0% in representative RET systems; higher contents used in some hybrid formulations	For 0.5–1.0% RET, representative data show penetration decreases of ∼24.3–31.3% and softening-point increases of up to ∼23.1%. Higher RET contents can increase crosslinking and gelation risk	62 and 67
Hybrid modifiers	SBS/EVA/RET, polymer–rubber, polymer-nanomaterial systems	System-specific; individual components commonly ∼0.5–5%	For hybrid CR/SBS and CR/SBS/EVA systems, addition of a crosslinking agent increased the softening point from 73.3 to 85.7 °C (+17%) and from 75.4 to 89.4 °C (+18.6%), respectively. Hybrid modification also reduced penetration and improved rheological resistance to permanent deformation	67 and 133
Polymer-assisted oxidative modification	Waste PE/PET-containing polymer waste	2–3%	Representative optimum: 2 wt% polymer waste at 270 °C for 4 h; the resulting product met OMB 100/130 requirements and showed thermal stability to approximately 350 °C	75

Overall, the classification of polymer modifiers should not be understood as a purely material-based list, but rather as a framework linking modifier chemistry, interaction mechanism, morphology development, and engineering performance. This broader classification is especially important for future-oriented binders, where high performance, sustainability, and multifunctionality are expected to be achieved simultaneously.^37,52^

Recently, increasing attention has been directed toward carbonaceous materials as sustainable and cost-effective alternatives or complementary modifiers to conventional polymer-modified bitumen (PMB). Among these materials, mechanochemically activated petroleum and coal microcoke have demonstrated considerable potential for enhancing the high-temperature performance of asphalt binders. Unlike elastomeric and plastomeric polymers, which improve pavement performance primarily through polymer swelling, network formation, and viscoelastic reinforcement, microcoke acts predominantly through a physical reinforcement mechanism by restricting molecular mobility within the bitumen matrix and improving stress transfer between the dispersed particles and the surrounding binder.

Ongarbayev *et al.*^66^ investigated the influence of mechanochemically activated petroleum and coal microcoke on BND 100/130 penetration-grade bitumen. High-energy ball milling significantly reduced the particle size of petroleum coke to approximately 8.3 µm, whereas coal coke reached an average size of 16.6 µm, substantially increasing the specific surface area available for interaction with the binder. XRD analysis further revealed that the activation process produced predominantly amorphous carbon with minor graphitic domains, while SEM observations confirmed the formation of flake-like particles capable of improving mechanical interlocking with the bitumen matrix. These structural modifications promoted improved dispersion and stronger interfacial adhesion between the carbon particles and the binder.

The improved morphology translated directly into enhanced rheological performance. Incorporation of only 1 wt% petroleum microcoke reduced the non-recoverable creep compliance (*J*_nr_ 3.2) from 4.07 to 2.08 kPa^−1^, indicating substantially greater resistance to permanent deformation under repeated loading. Simultaneously, the modified binder exhibited higher elastic recovery and lower stress sensitivity than the unmodified bitumen, while all modified binders satisfied the Superpave MSCR requirements for standard traffic conditions. Conventional penetration and softening-point tests also demonstrated increased stiffness, with the original BND 100/130 binder being upgraded to the BND 70/100 grade after modification.

From a mechanistic perspective, the reinforcement mechanism of microcoke differs fundamentally from that of conventional polymer modifiers. SBS, SIS, and other elastomers selectively absorb the maltene fraction of bitumen, swell, and subsequently form a continuous three-dimensional elastic network that enhances elasticity, fatigue resistance, and crack-healing capability.^37,50^ In contrast, microcoke particles remain dimensionally stable during mixing and reinforce the binder through particle-binder interactions, physical adsorption on asphaltene domains, mechanical interlocking, and restriction of molecular mobility. The enhanced specific surface area generated by mechanochemical activation further promotes stress transfer and limits viscous flow at elevated temperatures, thereby improving rutting resistance without relying on polymer network formation.

An additional advantage of microcoke is its industrial sustainability. Petroleum and coal coke are abundant by-products of petroleum refining and coal processing, making them economically attractive compared with virgin polymers. Mechanochemical activation is also a solvent-free and scalable process capable of producing highly reactive microdispersed particles while avoiding complex chemical functionalization. Consequently, microcoke represents a promising approach for valorizing industrial carbon waste within pavement engineering and contributes to circular economy strategies.

Nevertheless, despite these promising findings, carbonaceous modifiers cannot completely replace polymer modifiers in applications requiring high elastic recovery. The study clearly demonstrates that microcoke primarily increases stiffness and rutting resistance, whereas elastomeric polymers simultaneously improve elasticity, fatigue resistance, and crack resistance through network formation. Therefore, microcoke should be regarded as a complementary modifier rather than a direct substitute for SBS or other elastomers. Future research should focus on hybrid modification strategies combining mechanochemically activated microcoke with conventional polymers, where the rigid carbon particles provide enhanced stiffness and thermal stability while the polymer phase supplies elasticity and storage stability. Such synergistic systems may offer an attractive route toward high-performance, durable, and economically viable asphalt binders for future pavement applications.

Keyf^67^ demonstrated that combining elastomeric SBS, plastomeric EVA, and reactive ethylene terpolymer (RET) provides a synergistic enhancement of bitumen performance compared with using individual modifiers. While SBS contributes elasticity and EVA improves stiffness, RET chemically reacts with carboxylic groups in asphaltenes to form ester linkages, thereby enhancing polymer–bitumen compatibility and suppressing phase separation. FTIR analysis confirmed the formation of these chemical bonds, whereas conventional binder tests showed reduced penetration and ductility together with increased softening point, penetration index, elastic recovery, and viscosity. Notably, crosslinking became significant at approximately 2–2.5 wt% RET, indicating that reactive polymers can effectively stabilize polymer-modified binders while improving high-temperature performance. These findings demonstrate that integrating reactive and non-reactive polymers is an effective strategy for producing polymer-modified bitumen with balanced mechanical properties and improved storage stability.

Recent advances have expanded bitumen modification beyond conventional polymers to include carbon-based materials such as petroleum coke and carbon nanomaterials (CNMs), including graphene, graphene oxide, and carbon nanotubes.^68^ These materials enhance bitumen performance through mechanisms distinct from polymer modifiers, relying primarily on physical reinforcement, improved stress transfer, and strong interfacial interactions (*e.g.*, π–π stacking, hydrogen bonding, and covalent interactions) rather than polymer swelling and network formation. Petroleum coke has been reported to increase the softening point by 10–15 °C and improve rutting resistance by up to 30%, while graphene and CNTs significantly enhance stiffness, fatigue resistance, thermal stability, and aging resistance even at relatively low loadings. Furthermore, the utilization of coke, an abundant refinery by-product, offers an environmentally sustainable route for valorizing industrial waste and reducing the environmental footprint of pavement materials. Despite these advantages, challenges associated with the high cost of nanomaterials, dispersion homogeneity, increased binder viscosity, and large-scale implementation remain major obstacles to commercialization. Consequently, future research should focus on hybrid systems combining polymers with carbon nanomaterials or coke to exploit the elasticity of polymer networks together with the exceptional mechanical reinforcement and durability provided by carbon-based fillers, thereby enabling the development of high-performance and sustainable asphalt binders.

A major limitation of nanomaterial-modified asphalt is achieving homogeneous and reproducible nanoparticle dispersion. Experimental studies have shown that the performance of CNT- and graphene-modified binders is strongly dependent on the preparation and dispersion method, while nanoparticle agglomeration can limit the effectiveness of modification.^69,70^ For example, Amin *et al.* found that functionalization of MWCNTs did not necessarily improve their dispersion quality, whereas comparative processing studies demonstrated that the mixing route significantly affects CNT dispersion and the resulting asphalt performance.^69,70^ More recently, alternative processing approaches have been investigated specifically to suppress graphene agglomeration and improve its distribution within asphalt systems.^71^ These findings indicate that translating nanomodification from controlled laboratory preparation to reproducible large-scale production remains an important practical challenge.

The comparative analysis demonstrates (Table 2) that no modifier class provides a universal optimum across performance, compatibility, processability, sustainability, and cost.

Elastomeric modifiers such as SBS provide the strongest combination of elasticity and resistance to permanent deformation but require relatively intensive processing and may increase material cost and storage-stability concerns. Plastomers provide efficient high-temperature stiffening but can compromise low-temperature flexibility, whereas recycled thermoplastics offer similar reinforcement with potential sustainability and economic advantages at the expense of greater feedstock variability and compatibility challenges. Crumb rubber combines substantial viscoelastic modification with waste-tire valorization, although its relatively high dosage, viscosity, and processing requirements can offset part of its economic advantage. Reactive polymers provide improved compatibility and storage stability through chemical coupling at comparatively low dosage but require strict control of formulation and processing to avoid excessive crosslinking. Polymer-nanomaterial hybrids offer multifunctional enhancement but currently face the greatest barriers in terms of dispersion, cost, reproducibility, and scale-up. Therefore, modifier selection should be based not on improvement of a single binder property but on the required balance among climatic and traffic conditions, dominant pavement distress, processing capability, storage stability, sustainability objectives, and life-cycle cost.

The performance of polymer-modified bitumen is governed not only by the intrinsic chemistry of the modifier, but also by the processing route through which the polymer is incorporated into the binder. In practice, polymer modification is a coupled physicochemical and technological process in which blending temperature, shear intensity, mixing duration, polymer dosage, and the order of material addition collectively determine the extent of polymer dispersion, swelling, morphology development, and storage stability (Scheme 1).^37,76,110^ Among the available preparation routes, the wet process remains the most widely adopted for high-performance binder modification because the polymer is blended directly with hot bitumen under controlled conditions, allowing more intensive interaction between the modifier and the binder fractions.^50^ By contrast, in the dry process the polymer is introduced during asphalt mixture production, where its action occurs less exclusively within the binder phase and is therefore more dependent on aggregate-binder-polymer interactions, particle size, and mixture processing conditions.^39^ Although the dry route is often viewed as simpler and potentially more economical, especially for recycled polymers and waste-derived additives, it generally offers less control over polymer–bitumen interaction and may yield more variable performance outcomes than the wet process.^39,76^

**Scheme 1:**
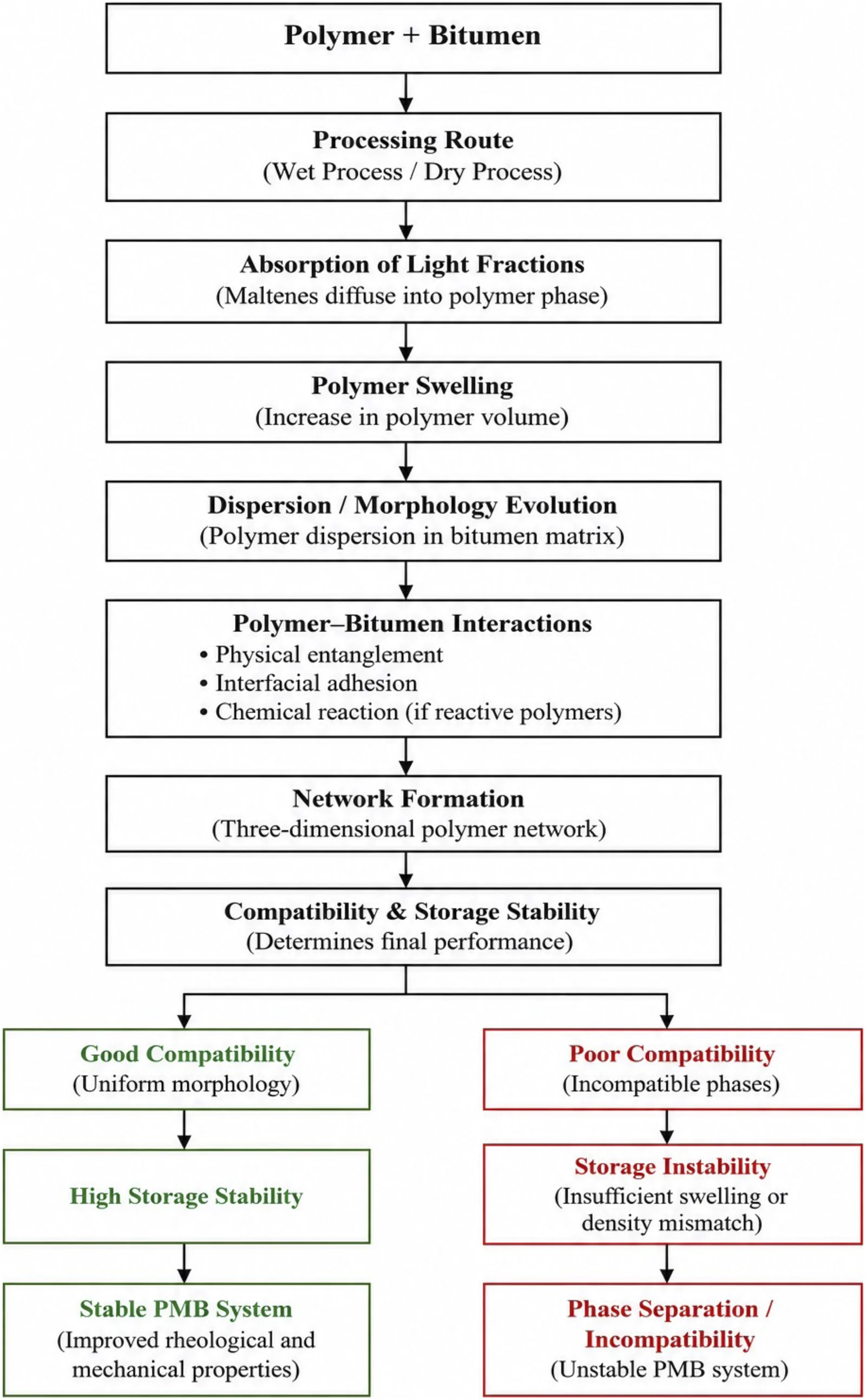
Overview of the physicochemical processes involved in polymer modification of bitumen. Following wet or dry processing, the diffusion of light bitumen fractions into the polymer phase promotes swelling, morphology evolution, and polymer–bitumen interactions, leading to the formation of a three-dimensional network. Depending on the degree of compatibility and storage stability, the modified binder either forms a stable polymer-modified bitumen (PMB) system or undergoes phase separation.

At the mechanistic level, one of the most important events during polymer modification is the absorption of light bitumen fractions, particularly the maltene-rich and aromatic components, by the polymer phase. This uptake softens the modifier, promotes diffusion-driven interaction, and initiates polymer swelling, which is widely recognized as a central step in the development of polymer-modified binder microstructure.^52,77^ As swelling progresses, the polymer expands in volume and progressively alters the internal phase balance of the binder, which in turn changes viscosity, elasticity, and resistance to permanent deformation.^37^ When the interaction is sufficiently favorable, these processes lead to the formation of a dispersed, co-continuous, or partially continuous polymer-rich network, which is one of the main structural explanations for the improved viscoelastic behavior of polymer-modified systems. Such network formation enhances the binder's ability to resist rutting at high temperature while preserving a greater degree of elastic recovery under repeated loading.^37,50^

However, the development of this beneficial structure is highly dependent on compatibility between the polymer and the base bitumen. If the polymer chemistry, molecular architecture, or processing conditions are poorly matched to the binder composition, the system may evolve into a strongly biphasic morphology in which polymer-rich and asphalt-rich phases separate during hot storage or transport.^56^ Recent multiscale analyses further show that phase separation is governed by the thermodynamic compatibility and molecular interactions between polymer and bitumen components, which subsequently control morphology evolution and storage stability.^78^ This phase separation is one of the most persistent limitations of polymer-modified bitumen, particularly in polyolefin-rich systems and some recycled-plastic formulations, because it leads to non-uniform binder properties, poor storage stability, and unreliable field performance.^52^ For this reason, current PMB design increasingly relies on compatibilizers, reactive polymers, crosslinkers, and carefully controlled processing strategies to improve interfacial stability and suppress phase segregation during storage and service.^52^

The mechanisms underlying modifier-bitumen interactions can be investigated using complementary chemical, thermal, microstructural, and rheological techniques. FTIR spectroscopy provides information on changes in functional groups and can therefore help distinguish physical mixing from possible chemical interactions, while SEM, fluorescence microscopy, and AFM provide information on modifier dispersion, phase morphology, and interfacial structure. Thermal methods such as DSC, DTA, and thermogravimetric analysis provide additional evidence of changes in thermal transitions and stability, whereas rheological techniques such as DSR and MSCR relate these structural changes to the viscoelastic and deformation behavior of the binder.^111^

A recent study by Prysiazhnyi *et al.*^79^ illustrates the value of combining several analytical methods for mechanistic interpretation. Using DTA/DTG, FTIR, and SEM, the authors investigated interactions between long-term stored oxidized lignin waste and bitumen. FTIR revealed the disappearance of absorption bands associated with reactive methoxyl groups and organometallic structures after lignin incorporation, while SEM indicated changes in the morphology of the modified binder. Together with the thermal-analysis results, these observations were interpreted as evidence that lignin participates in chemical interactions with bitumen under the applied modification conditions. However, the authors also identified rheological characterization, low-temperature testing, aging, and storage-stability evaluation as necessary directions for further investigation, demonstrating that chemical and morphological evidence alone is insufficient for a comprehensive assessment of modifier performance.^79^

The relative importance of these mechanisms varies with polymer chemistry and molecular architecture. Elastomeric polymers, including SBS and styrene-butadiene rubber (SBR), are among the most widely used modifiers in pavement engineering. Their primary mechanism involves the development of an elastic network capable of storing and recovering deformation energy under traffic loading. SBS-modified binders exhibit enhanced elasticity, improved rutting resistance at high temperatures, and increased fatigue life due to the formation of a continuous polymer network within the bitumen matrix.^80,81^

The swollen SBS particles create a reinforcing structure that restricts molecular mobility and increases the complex modulus of the binder. Consequently, the modified binder demonstrates improved resistance to permanent deformation while maintaining flexibility at intermediate and low temperatures. Furthermore, the elastic recovery of SBS-modified bitumen contributes to reduced crack propagation and improved long-term pavement durability.^81,82^

Unlike elastomers, plastomeric polymers such as polyethylene (PE), polypropylene (PP), and ethylene-vinyl acetate (EVA) primarily enhance stiffness and strength rather than elasticity. These polymers increase the rigidity of the binder by forming crystalline or semi-crystalline domains within the bituminous matrix.^83^

The mechanism of plastomer modification involves the dispersion of polymer particles throughout the binder, leading to increased viscosity and improved load-bearing capacity. EVA, for example, combines both elastomeric and plastomeric characteristics due to the presence of flexible vinyl acetate groups and crystalline ethylene segments. As a result, EVA-modified binders exhibit enhanced rutting resistance, improved storage stability, and satisfactory low-temperature performance.^84^ However, excessive plastomer content may reduce flexibility and increase susceptibility to thermal cracking, necessitating careful optimization of polymer concentration.^83,84^

Reactive polymers represent a more advanced approach to bitumen modification. Unlike conventional physical blending, reactive modification involves chemical reactions between functional groups on the polymer and polar components of bitumen. Polymers containing epoxy, maleic anhydride, isocyanate, or silane functionalities can form covalent bonds with bitumen molecules, resulting in enhanced compatibility and storage stability.^9^

The formation of chemical crosslinks restricts phase separation during storage and improves aging resistance by stabilizing the binder microstructure. Reactive modification also increases resistance to oxidative degradation and improves mechanical performance under long-term service conditions.^85,86^

Recent advances in nanotechnology have introduced a new generation of polymer-modified binders incorporating nanoclays, graphene nanoplatelets, carbon nanotubes (CNTs), nanosilica, and other nanostructured materials. These nanomaterials provide reinforcement through their exceptionally high surface area and strong interfacial interactions with both the polymer and bitumen phases.^87,88^

Nanoclays can form exfoliated or intercalated structures within the binder, creating tortuous diffusion pathways that improve thermal stability and aging resistance. Carbon nanotubes and graphene derivatives enhance mechanical properties by facilitating stress transfer at the nanoscale and increasing the stiffness of the polymer network.^87,89^ Furthermore, hybrid systems combining polymers with nanomaterials often exhibit synergistic effects, resulting in superior rutting resistance, fatigue performance, and moisture damage resistance compared with conventional PMBs.^88,89^

Growing environmental concerns have accelerated the use of recycled plastics, crumb rubber, bio-based polymers, and waste-derived modifiers in asphalt technology. Recycled polyethylene, polypropylene, and polyethylene terephthalate (PET) can improve high-temperature performance through mechanisms similar to those observed in virgin plastomers.^90^ Crumb rubber modification involves the absorption of aromatic fractions from bitumen, causing rubber swelling and the formation of a viscoelastic network that enhances crack resistance and pavement durability.^91^

Bio-based modifiers derived from vegetable oils, lignin, biomass residues, and waste cooking oils introduce renewable functional groups capable of interacting with bitumen molecules. These modifiers improve sustainability while reducing dependence on petroleum-derived materials and lowering the environmental footprint of road construction.^92^

Despite the significant advantages of polymer modification, several challenges remain. The primary issue is the thermodynamic incompatibility between many polymers and bitumen components, which can lead to phase separation during storage and transportation.^77,93^ Differences in density, polarity, and molecular weight between the polymer and bitumen fractions frequently result in unstable morphologies. Moreover, aging processes involving oxidation, volatilization, and UV degradation can alter the polymer network and diminish long-term performance.^82^

Future research is increasingly focused on compatibilizers, reactive modification strategies, nanostructured additives, and machine-learning-assisted formulation design to optimize polymer–bitumen interactions and ensure durable, sustainable pavement materials for next-generation transportation infrastructure.

## 4. Performance of polymer-modified bitumen in road construction

The primary engineering advantage of polymer-modified bitumen in road construction lies in its ability to improve binder performance under demanding service conditions. At high temperatures, polymer modification generally increases resistance to permanent deformation and rutting, while at intermediate temperatures it enhances elastic recovery and fatigue performance under repeated loading. Earlier and more recent reviews consistently indicate that polymer-modified binders provide a more favorable balance between stiffness and elasticity than conventional binders, which is essential for pavements subjected to heavy traffic and climatic variability.^37,50^

The performance improvement of polymer-modified bitumen, however, depends strongly on the type of modifier used. Elastomeric modifiers such as SBS generally provide better elasticity, enhanced fatigue resistance, and improved recoverable deformation, whereas plastomeric modifiers such as EVA and polyethylene are more often associated with increased stiffness and rutting resistance. Comparative studies on SBS- and EVA-modified binders have shown that differences in microstructure and polymer dispersion lead to distinct rheological and mechanical responses, confirming that binder morphology is directly linked to pavement performance.^83^

In practical pavement engineering, the benefits of polymer modification extend beyond binder-scale properties to asphalt mixture performance. Reviews on asphalt mixtures modified with polymeric waste indicate that both wet and dry processing routes can improve rutting resistance, stiffness, and, in many cases, overall pavement durability, although outcomes remain highly dependent on polymer type, dosage, and processing method.^39^ Similarly, crumb-rubber-modified systems have been widely reported to improve deformation resistance, fatigue-related behavior, and service sustainability by reusing end-of-life tires in road materials.^84^

Despite these advantages, the full field performance of polymer-modified bitumen still depends on compatibility, storage stability, aging resistance, and construction practice. Some modifiers deliver substantial gains in laboratory testing but remain sensitive to blending conditions or long-term thermal-oxidative degradation. For this reason, current research increasingly emphasizes performance-based binder design, improved processing control, and the integration of sustainable modifiers that can simultaneously enhance engineering properties and support long-life pavement strategies.^37,39^

Lesueur^85^ demonstrated that polymer-modified bitumen emulsions (PMBEs) provide an effective route for combining the mechanical advantages of polymer-modified binders with the environmental benefits of cold asphalt technologies. Unlike conventional hot-mix PMBs, PMBEs can be produced either by emulsifying a pre-modified binder or by incorporating polymer latex before or after emulsification, allowing greater flexibility in formulation while maintaining improved rheological properties after emulsion breaking.

A key finding is that the modification route strongly influences the final microstructure of the binder. Latex-modified emulsions can form a continuous polymer-rich network after breaking with only 2–3 wt% polymer, whereas conventional hot-process PMBs generally require polymer contents above 6 wt% to achieve comparable network continuity. This unique morphology enhances elasticity, cohesion, and aggregate retention while improving resistance to rutting and traffic-induced damage.

The study also highlights that successful PMBE production requires careful control of emulsification conditions because polymer modification increases binder viscosity and alters droplet deformation during milling, leading to coarser particle-size distributions and reduced storage stability if processing parameters are not optimized. Consequently, emulsion formulation—including emulsifier dosage, polymer type, and drying conditions—is as critical as polymer selection itself.

Field applications further confirm that PMBEs significantly improve the performance of chip seals and microsurfacings by increasing aggregate retention, reducing wet abrasion loss, and extending pavement durability under heavy traffic. These findings demonstrate that PMBEs represent a sustainable alternative to conventional hot polymer-modified binders, providing enhanced pavement performance while reducing energy consumption and greenhouse gas emissions associated with asphalt production.

As summarized in Table 3, no single characterization technique is sufficient to comprehensively evaluate polymer-modified bitumen; therefore, complementary rheological, chemical, thermal, and microstructural analyses are generally required.

**Table 3:** Characterization techniques for evaluating the properties and performance of polymer modified bitumen modified bitumen

Method	Parameter measured	Main output	Relevance to PMB
Penetration test	Consistency/hardness	Penetration value	Evaluates binder hardness and relative softness after polymer modification
Softening point test	Thermal susceptibility	Softening temperature	Assesses high-temperature resistance and thermal stability
Rotational viscosity	Flow behavior at high temperature	Viscosity value	Determines workability, mixing, and compaction characteristics
Dynamic shear rheometer (DSR)	Viscoelastic properties	Complex modulus (G*), phase angle (*δ*)	Evaluates rutting and fatigue resistance of modified binders
Bending beam rheometer (BBR)	Low-temperature stiffness	Creep stiffness (*S*), *m*-value	Assesses thermal cracking resistance at low temperatures
Multiple stress creep recovery (MSCR)	Recovery and permanent deformation	Non-recoverable creep compliance, percent recovery	Measures rutting resistance and elastic recovery under repeated loading
Fourier transform infrared spectroscopy (FTIR)	Chemical structure and functional groups	Characteristic absorption bands, oxidation indices	Identifies polymer–bitumen interactions and aging-related chemical changes
Thermogravimetric analysis (TGA)	Thermal stability and degradation	Weight loss profile, degradation temperature	Evaluates thermal decomposition behavior of modified binders
Differential scanning calorimetry (DSC)	Thermal transitions	Glass transition, melting, crystallization behavior	Provides information on thermal events and polymer compatibility
Fluorescence microscopy	Morphology and phase distribution	Polymer-rich domain images	Visualizes dispersion, phase separation, and storage stability
Scanning electron microscopy (SEM)	Surface morphology	Microstructural images	Examines dispersion of modifiers and structural heterogeneity
Atomic force microscopy (AFM)	Surface topography and nanoscale morphology	Surface roughness, phase contrast	Supports structure–property analysis at nanoscale
Storage stability test	Phase separation tendency	Difference in softening point or rheological response between top and bottom sections	Assesses stability of polymer-modified binders during hot storage
Aging simulations (RTFO/PAV)	Short-term and long-term aging behavior	Aged binder properties	Evaluates durability and performance retention after oxidative aging

## 5. Microstructure and morphology of modified bitumen

The performance of polymer-modified bitumen (PMB) is strongly governed by its microstructure and morphology, which determine the distribution of stresses, viscoelastic response, storage stability, aging behavior, and overall pavement performance. Unlike conventional bitumen, which consists of a complex colloidal system of saturates, aromatics, resins, and asphaltenes (SARA fractions), PMB exhibits a multiphase structure in which the polymer and bitumen phases interact through physical and, in some cases, chemical mechanisms.^86^ The resulting morphology depends on numerous factors, including polymer type, concentration, molecular weight, blending conditions, and the chemical composition of the base bitumen.^87^

The colloidal model of bitumen describes asphaltene micelles dispersed within a continuous maltene phase stabilized by resins. The introduction of polymers significantly alters this microstructure by changing the interactions among the SARA fractions and modifying the spatial organization of the dispersed phases.^85^ Depending on polymer concentration and compatibility, the microstructure may evolve from isolated polymer-rich domains dispersed in a bitumen-rich matrix to a continuous polymer network encompassing the entire binder system.^86,88^

### 5.1 Phase structure and morphological development

The morphology of PMB commonly comprises polymer-rich and bitumen-rich phases whose relative continuity depends on polymer concentration and compatibility. During blending, aromatic components of bitumen diffuse into the polymer phase and promote swelling. At relatively low polymer contents, swollen polymer-rich domains remain dispersed within a continuous bitumen-rich phase; with increasing polymer concentration and swelling, these domains may interconnect and eventually undergo phase inversion to form a continuous or co-continuous polymer-rich network.^90,91^ This morphological transition is closely associated with enhanced elastic recovery and resistance to permanent deformation.

The morphology of plastomer-modified binders differs substantially from that of elastomer-modified systems. Polymers such as polyethylene (PE), polypropylene (PP), and ethylene-vinyl acetate (EVA) tend to form dispersed crystalline or semi-crystalline domains that reinforce the bitumen matrix through physical stiffening mechanisms. These structures increase stiffness and rutting resistance but may reduce flexibility at low temperatures if excessive polymer concentrations are employed.^36,92^

Importantly, improved rheological performance does not necessarily indicate good thermodynamic compatibility or long-term storage stability. Polymer-modified bitumen may exhibit enhanced stiffness and elasticity while remaining a thermodynamically unstable multiphase system, leading to polymer-rich and bitumen-rich phase separation during high-temperature storage.^78,94^ Compatibility and phase stability depend strongly on base-binder composition, polymer chemistry and architecture, modifier concentration, swelling behavior, and processing conditions; therefore, rheological measurements should be complemented by storage-stability and morphological characterization when evaluating PMB systems.

### 5.2 Microscopic characterization techniques

Understanding PMB morphology requires advanced characterization methods capable of visualizing phase distribution and polymer–bitumen interactions. Fluorescence microscopy is one of the most widely employed techniques because many polymers fluoresce while bitumen remains relatively dark. This contrast allows direct observation of polymer-rich domains and network formation.^90^

Optical microscopy and laser scanning confocal microscopy have also been extensively utilized to investigate polymer dispersion, swelling behavior, and phase continuity. These techniques reveal that the degree of polymer dispersion strongly influences storage stability and mechanical performance.^88,90^

Scanning electron microscopy (SEM) provides detailed information regarding surface morphology and fracture characteristics of modified binders. SEM analyses frequently demonstrate more homogeneous and interconnected structures in polymer-modified systems compared with conventional bitumen.^91^ Furthermore, atomic force microscopy (AFM) has emerged as a powerful tool for investigating nanoscale morphology. AFM studies reveal characteristic ‘bee-like’ surface structures in bitumen and demonstrate how polymer addition can modify the distribution and organization of nanoscale morphological features.^36,92^

### 5.3 Nanostructured morphology in advanced PMBs

Recent developments in nanotechnology have introduced new opportunities for tailoring PMB microstructure. Nanomaterials such as nanoclays, graphene nanoplatelets, carbon nanotubes (CNTs), nanosilica, and layered silicates create hierarchical microstructures with enhanced interfacial interactions.^42,45,95^

Nanoclays typically form intercalated or exfoliated structures within the polymer–bitumen matrix, increasing tortuosity and restricting molecular mobility. These effects improve thermal stability, aging resistance, and mechanical performance.^95^ Similarly, graphene derivatives and CNTs establish reinforcing nanonetworks that facilitate stress transfer across interfaces and enhance stiffness, fracture resistance, and durability.^42,45^

Hybrid systems incorporating both polymers and nanomaterials frequently exhibit synergistic effects. Hybrid systems incorporating both polymers and nanomaterials may exhibit synergistic effects when adequate dispersion and interfacial compatibility are achieved. Well-dispersed nanomaterials can reinforce polymer–bitumen interfaces and modify polymer-rich phase morphology; however, agglomeration can reduce these benefits and compromise reproducibility. As a result, hybrid PMBs often display superior storage stability, rutting resistance, and fatigue performance compared with conventional polymer-modified binders.^42^

### 5.4 Influence of morphology on performance

The relationship between morphology and performance is one of the most critical aspects of PMB design. Continuous polymer networks generally provide greater elasticity, higher recovery after deformation, and enhanced fatigue resistance.^90,91^ Conversely, poorly dispersed polymer domains or phase-separated systems often exhibit inferior storage stability and reduced long-term performance.

Aging processes also influence PMB morphology. Oxidative aging causes changes in the chemical composition of bitumen, generally increasing the relative contribution of heavier and more polar fractions and altering phase structure. In PMBs, aging may additionally affect polymer integrity and polymer–bitumen interactions, resulting in morphological evolution and increased embrittlement.^96,97^ Consequently, understanding the coupled chemical and morphological evolution of PMBs during aging is essential for predicting long-term pavement durability.

The incorporation of recycled polymers, bio-based modifiers, and nanomaterials introduces additional complexity because feedstock variability, interfacial incompatibility, and particle agglomeration can produce heterogeneous and less reproducible morphologies. Therefore, future PMB design should couple morphological characterization with rheological, storage-stability, and aging analyses rather than assuming that improved dispersion alone guarantees superior long-term performance.

As summarized in Table 4, each characterization technique provides complementary information on the morphology of polymer-modified bitumen.

**Table 4:** Microstructural features of polymer-modified bitumen revealed by different characterization techniques

Technique	Information obtained	Influence on performance
Fluorescence microscopy	Polymer dispersion	Storage stability
SEM	Phase morphology	Crack resistance
AFM	Bee structures	Aging
Raman	Chemical mapping	Compatibility
X-ray CT	3D network	Durability

Therefore, combining microscopic, spectroscopic, and rheological analyses enables a more comprehensive understanding of the relationships between microstructure and pavement performance.

## 6. Sustainability and the future of road construction

The global transportation sector is undergoing a profound transformation driven by increasing environmental concerns, resource scarcity, climate change mitigation targets, and the need for resilient infrastructure systems. Road construction, which consumes enormous quantities of raw materials and energy, has become a focal point for sustainability initiatives aimed at reducing greenhouse gas (GHG) emissions, minimizing waste generation, conserving natural resources, and extending pavement service life.^41,46,47^ In this context, polymer-modified bitumen (PMB) and emerging sustainable pavement technologies are playing a crucial role in shaping the next generation of environmentally responsible transportation infrastructure.

### 6.1 Environmental challenges in conventional road construction

Traditional asphalt pavement production relies heavily on petroleum-derived bitumen, virgin aggregates, and energy-intensive manufacturing processes. The extraction, transportation, and processing of these materials contribute significantly to carbon emissions and environmental degradation.^41^ Furthermore, premature pavement deterioration leads to frequent maintenance and rehabilitation activities, resulting in additional energy consumption, traffic disruptions, and economic losses.^46^

Life-cycle assessment (LCA) studies have demonstrated that pavement sustainability must be evaluated throughout the entire life cycle, including raw material extraction, construction, operation, maintenance, and end-of-life management.^41,47^ Consequently, modern pavement engineering increasingly focuses on reducing environmental impacts while simultaneously improving long-term performance and durability.

### 6.2 Circular economy and waste valorization

One of the most promising approaches toward sustainable road construction is the implementation of circular economy principles. Instead of treating waste materials as environmental burdens, modern pavement technologies increasingly utilize recycled resources as valuable engineering materials.^48^

Waste plastics, including polyethylene (PE), polypropylene (PP), polyethylene terephthalate (PET), and mixed plastic wastes, have attracted considerable attention as bitumen modifiers. These materials improve rutting resistance and stiffness while simultaneously reducing plastic pollution and landfill disposal.^98,99^ Numerous studies have reported that appropriately formulated recycled-plastic-modified asphalt mixtures can achieve mechanical performance comparable to, and in some cases higher than, conventional asphalt mixtures.^99^

Similarly, crumb rubber obtained from end-of-life tires has become one of the most successful examples of waste valorization in pavement engineering. The incorporation of crumb rubber improves elasticity, fatigue resistance, crack resistance, and noise reduction while providing a sustainable disposal route for millions of waste tires generated annually worldwide.^100^

In addition, reclaimed asphalt pavement (RAP) has emerged as a key component of sustainable pavement management. RAP utilization reduces the demand for virgin aggregates and bitumen while significantly lowering construction costs and environmental impacts.^101^ Nevertheless, waste origin alone does not guarantee environmental benefit, because additional sorting, pretreatment, transportation, elevated processing temperatures, and compatibility-control measures may partly offset the benefits of material diversion.

### 6.3 Bio-based and renewable modifiers

The replacement of petroleum-derived materials with renewable alternatives is another important pathway toward sustainable road construction. Bio-based modifiers derived from vegetable oils, lignin, biomass residues, algae, and waste cooking oils have been investigated extensively as potentially lower-impact alternatives to conventional asphalt modifiers.^50,102^

Lignin, one of the most abundant natural polymers on Earth, has demonstrated considerable potential as a partial bitumen replacement due to its aromatic structure and antioxidant properties. Studies have shown that lignin-modified binders can improve aging resistance and reduce dependence on fossil resources.^50^

Bio-oils derived from biomass pyrolysis and agricultural waste streams have also been explored as rejuvenators and modifiers for aged binders. These renewable materials contribute to reduced carbon footprints while promoting the utilization of sustainable feedstocks.^102^ Although challenges related to long-term durability and storage stability remain, bio-based asphalt technologies are expected to play an increasingly important role in future pavement systems.

### 6.4 Life-cycle and performance comparison of bitumen modifiers

While the technical performance of representative modification strategies is compared in Table 6, assessment of their sustainability requires simultaneous consideration of technical benefits and life-cycle environmental impacts.

A consistent comparison of virgin polymers, recycled polymers, bio-based modifiers, and nanomaterials requires consideration of both technical performance and life-cycle environmental impacts. Virgin polymers such as SBS and EVA provide well-established improvements in rutting resistance, elasticity, and durability, but their production relies on fossil-derived feedstocks and additional processing energy.^58,103^ Recycled polymers can provide comparable technical benefits while simultaneously diverting waste from disposal; however, their environmental advantage depends strongly on waste collection, sorting, reprocessing, transportation, mixing temperature, and service-life extension.^103,104^ For example, a cradle-to-grave assessment of recycled-plastic-modified asphalt reported a projected 14–65% increase in surface-layer service life, depending on pavement structure and traffic, which extended rehabilitation intervals, although increased vehicle emissions associated with pavement roughness offset part of the environmental benefit.^105^ Bio-based modifiers offer the potential to reduce dependence on petroleum-derived materials, but their performance and environmental benefits depend on feedstock type, processing route, dosage, and the extent to which they replace conventional binder; moreover, substantial inconsistencies in system boundaries and omission of maintenance, use, and end-of-life stages in existing sustainability studies limit direct comparison.^106^ Nanomaterials can achieve significant rheological enhancement at relatively low dosages, but their production may introduce additional energy and environmental burdens.^107^ For instance, a production-stage LCA of nano-silica-modified asphalt reported a global warming potential of 7.446 × 10^3^ kg CO_2_-eq per functional unit, compared with 7.419 × 10^3^ kg CO_2_-eq for the corresponding conventional mixture, representing an approximately 0.4% increase.^107^ Thus, no modifier class can presently be identified as universally superior on both technical and environmental grounds. Meaningful comparisons should use a common functional unit and system boundary and simultaneously consider modifier dosage, processing energy, performance improvement, service-life extension, maintenance frequency, transportation, and end-of-life scenarios.^103,106,107^ The lack of harmonized life-cycle inventories and performance-normalized LCA data therefore remains an important research gap, particularly for emerging bio-based and nanomaterial-modified systems.

As shown in Table 5, improvements in technical performance do not necessarily correspond to reductions in life-cycle environmental impacts.

**Table 5:** Technical and life-cycle comparison

Modifier class	Representative system	Technical/performance evidence	Environmental/LCA evidence	Critical consideration	Reference
Virgin polymer	Linear SBS (SBS-L), BIT 70/100, 2–7 wt%	SHRP critical temperature increased from 65.1 °C (base binder) to 68.0, 73.9, 85.0, 91.6, and 100.2 °C at 2, 3, 4, 5, and 7% SBS-L, respectively	Virgin SBS is fossil-derived; production and processing contribute additional material and energy burdens compared with some recycled-polymer alternatives	Strong high-temperature improvement, but higher polymer contents may increase cost and aggravate compatibility and storage-stability problems	58 and 103
Virgin polymer	Radial SBS (SBS-R), BIT 70/100, 2–4 wt%	SHRP critical temperature increased from 65.1 °C to 67.7, 75.8, and 80.6 °C at 2, 3, and 4% SBS-R, respectively	Environmental considerations are similar to other virgin SBS systems and depend on polymer production, dosage, and asphalt processing requirements	Molecular architecture affects performance; SBS concentration alone is therefore insufficient for cross-study comparison	58
Recycled polymer	Reprocessed/recycled-plastic-modified asphalt	A cradle-to-grave pavement assessment projected a 14–65% increase in surface-layer service life, depending on pavement structure and traffic	Reprocessed plastics can reduce demand for virgin polymer and divert plastic waste; reported benefits depend on collection, sorting, reprocessing, transport, mixing temperature, and service-life assumptions	Recycled origin does not automatically ensure lower life-cycle impacts; processing and transportation can offset part of the benefit	103 and 105
Bio-based modifier	Lignin, 25% replacement of bituminous binder	Partial binder replacement can reduce petroleum-bitumen demand; engineering performance depends on lignin source, dosage, processing, and binder formulation	A cradle-to-gate assessment reported a 5.72% reduction in global warming potential at 25% lignin substitution	Environmental benefits are strongly dependent on feedstock and LCA assumptions; cradle-to-gate results do not capture the complete use and end-of-life stages	106
Nanomaterial	Nano-silica-modified asphalt mixture	Nanomaterials can improve rheological and mixture properties at relatively low dosages, but effectiveness depends strongly on dispersion and formulation	Production-stage GWP was reported as 7.446 × 10^3^ kg CO_2_-eq/FU for nano-silica-modified asphalt *versus* 7.419 × 10^3^ kg CO_2_-eq/FU for conventional asphalt (≈0.4% higher)	Technical improvement does not necessarily reduce environmental impact; nanoparticle production, dispersion, safety, cost, and scale-up should be considered	107

The available evidence also demonstrates that comparisons among modifier classes remain strongly dependent on the selected functional unit, system boundary, processing conditions, and assumed pavement service life. Therefore, future studies should combine performance-normalized LCA with long-term pavement data to enable more reliable comparisons among alternative modification strategies.

### 6.5 Energy-efficient pavement technologies

Sustainability in road construction extends beyond material selection to include energy-efficient construction methods. Warm-mix asphalt (WMA) technology has emerged as one of the most significant innovations in this field. Unlike conventional hot-mix asphalt, WMA allows mixing and compaction at temperatures 20–40 °C lower, thereby reducing fuel consumption, greenhouse gas emissions, and occupational exposure to fumes.^13,119^

Numerous studies have demonstrated that WMA technologies can reduce energy consumption by up to 30% while maintaining comparable mechanical performance to traditional asphalt mixtures.^13,119^ The combination of WMA with polymer-modified binders, recycled materials, and bio-based additives further enhances environmental benefits and contributes to more sustainable pavement systems.

### 6.6 Self-healing and long-life pavements

Future road construction increasingly emphasizes durability rather than frequent maintenance. Self-healing asphalt materials represent an innovative approach to extending pavement service life through autonomous crack repair mechanisms.^31,32,130^ Various self-healing technologies have been developed, including induction heating, microwave heating, encapsulated rejuvenators, and conductive nanomaterials.

Wei *et al.* (2023) systematically reviewed the use of dynamic chemistry as an emerging strategy for self-healing polymer-modified asphalt.^130^ The authors distinguished conventional externally assisted healing approaches, including microcapsules, hollow fibers and induction heating, from intrinsic self-healing based on reversible polymer networks. They highlighted dynamic covalent and non-covalent interactions that allow damaged polymer networks to reorganize and reform under appropriate stimuli, enabling repeated healing rather than one-time release of a healing agent.

Importantly, Wei *et al.* identified epoxy, polyurethane and rubber as major polymer platforms for this technology and discussed the incorporation of reversible bonds such as imine, disulfide, ester, borate ester and Diels–Alder linkages.^130^ For example, lignin-based dynamic epoxy asphalt containing exchangeable ester bonds showed a tensile strength of 1.605 MPa and elongation at break of 382.38%, while thermal treatment enabled repeated mechanical recovery. Another ester-exchange epoxy asphalt achieved >70% mechanical-strength recovery after treatment at 180 °C for 20 min. Diels–Alder-based epoxy asphalt also showed regeneration with only about 2% tensile-strength loss after regeneration, although the authors noted that long-term performance over multiple regeneration cycles remains insufficiently established.

By restoring microcracks before they evolve into major failures, self-healing systems can substantially increase pavement lifespan while reducing maintenance interventions and resource consumption (Fig. 5).^131^ This approach aligns closely with sustainability goals by maximizing infrastructure longevity and minimizing life-cycle environmental impacts.

**Fig. 5:**
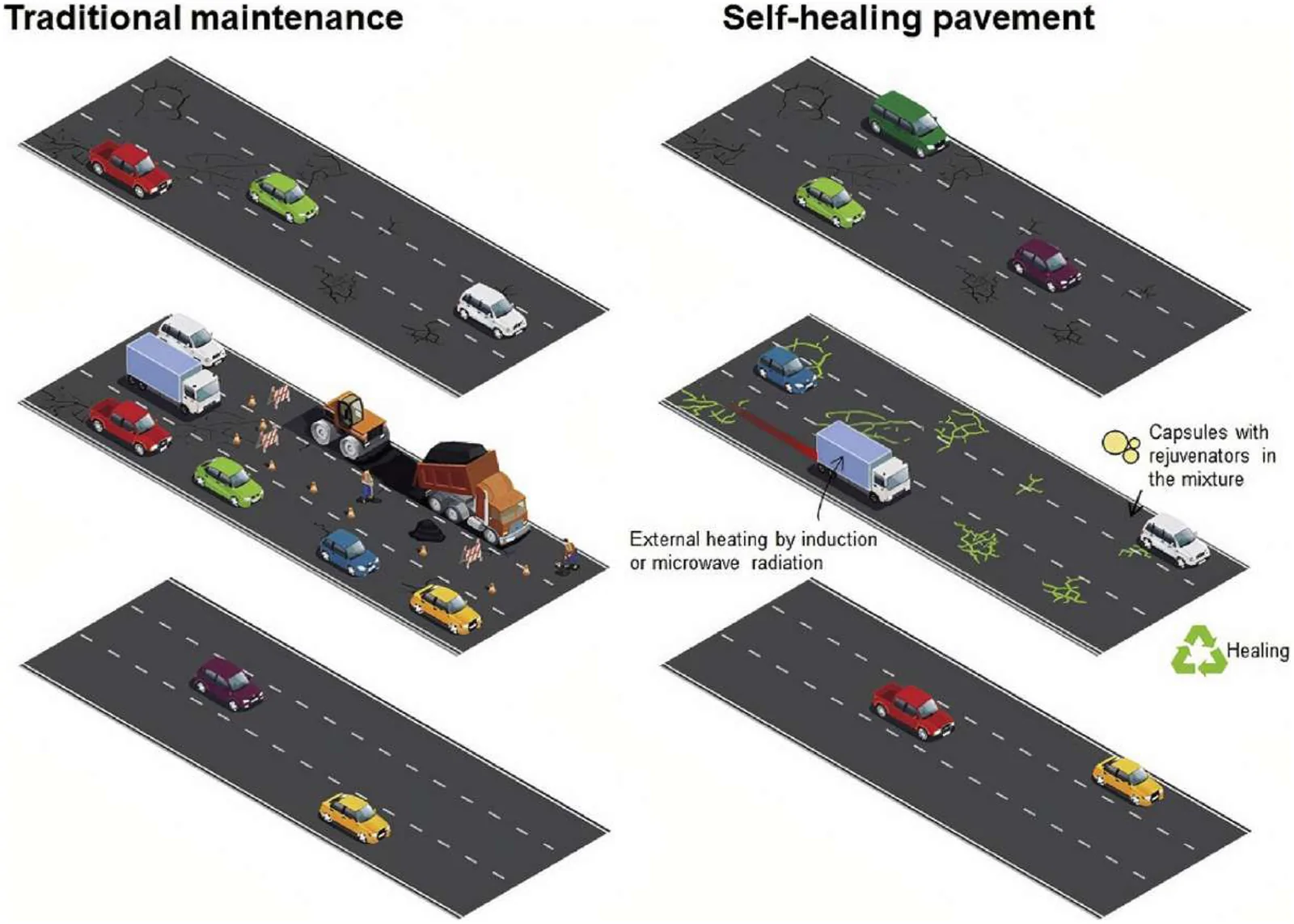
Comparison of traditional pavement repair and self-healing pavement processes. Adapted from Nalbandian *et al.*^131^

Norambuena-Contreras *et al.* (2022) developed sodium-alginate-based polynuclear capsules containing waste cooking oil (WCO) as a sustainable rejuvenating system for asphalt self-healing (Fig. 6). The capsules showed approximately 90% encapsulation efficiency, adequate mechanical and thermal stability, and the ability to release WCO under mechanical damage. Most importantly, the released WCO diffused into aged bitumen, reduced its viscosity, and achieved a maximum microcrack-healing efficiency of about 70% after 85 min, demonstrating the potential of combining a renewable biopolymer with a waste-derived rejuvenator for more sustainable self-healing asphalt.^132^

**Fig. 6:**
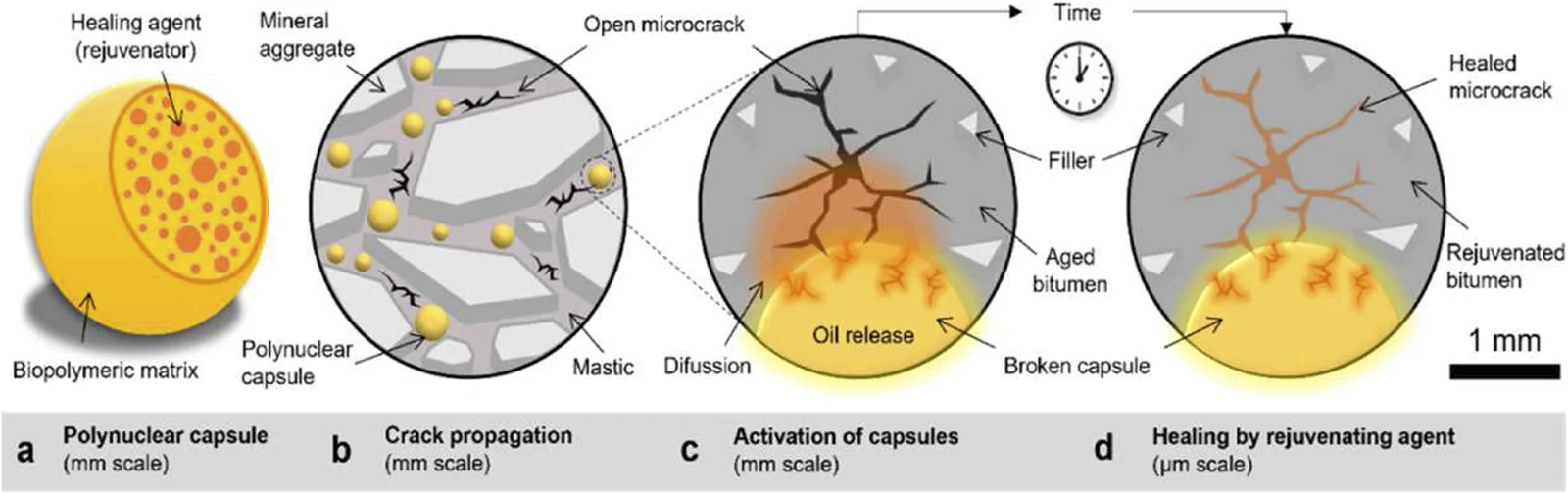
Schematic representation of the self-healing mechanism in asphalt using encapsulated rejuvenators: (a) encapsulated healing agent, (b) crack propagation, (c) capsule rupture and rejuvenator release, and (d) diffusion of the rejuvenator and crack healing. Adapted from Nalbandian *et al.*^131^ Norambuena-Contreras, J. *et al.*^132^

### 6.7 Smart and digital pavement systems

The digital transformation of transportation infrastructure is creating new opportunities for sustainable pavement management. Smart pavements equipped with embedded sensors, Internet of Things (IoT) technologies, wireless communication systems, and structural health monitoring networks can provide real-time information regarding pavement condition, traffic loads, temperature distribution, and damage evolution.^46^

These technologies enable predictive maintenance strategies that optimize resource allocation and prevent premature pavement deterioration. Furthermore, machine learning and artificial intelligence (AI) are increasingly employed to accelerate asphalt mixture design, predict pavement performance, and support decision-making processes.^77^ The integration of advanced materials with digital technologies represents a key element of future intelligent transportation systems.

### 6.8. Carbon neutrality and future perspectives

Achieving carbon-neutral transportation infrastructure is becoming a global priority. Future research is expected to focus on reducing greenhouse gas emissions through the increased use of recycled and renewable materials, low-temperature construction technologies, carbon capture strategies, and longer-lasting pavement systems.^113,114^

Polymer-modified bitumen will continue to play a central role in this transition by enhancing pavement durability and reducing life-cycle environmental impacts. Simultaneously, advances in nanotechnology, bio-based materials, circular economy concepts, digital engineering, and AI-driven optimization will further transform the road construction industry.

The future of sustainable road construction lies in the development of multifunctional pavement systems that combine high performance, environmental responsibility, resource efficiency, and intelligent monitoring capabilities. Such systems will be essential for meeting the growing demands of modern transportation networks while supporting global sustainability objectives.

Despite these advances, the emerging technologies discussed above differ substantially in their level of technological maturity and field validation. Waste-plastic modification has progressed beyond laboratory evaluation, with full-scale field trials demonstrating its practical applicability, although field performance may be affected by construction-related factors such as compaction and interlayer bonding.^115^ Among self-healing approaches, induction heating has reached field demonstration, including pilot pavement sections, whereas microwave heating and microcapsule-based systems remain predominantly laboratory-scale technologies with limited or no systematic field validation.^116^ Smart pavement systems based on embedded and wireless sensors have also been experimentally investigated for pavement structural-health monitoring, but challenges related to sensor durability, power supply, data transmission, and long-term implementation remain ref. 46. More advanced concepts combining multifunctional modification, autonomous healing, sensing, and adaptive pavement behavior remain largely at the experimental or conceptual stage. Consequently, long-term field monitoring, standardized performance assessment, scalability, and cost-effectiveness remain important requirements before widespread implementation of these emerging technologies.

As summarized in Table 6, sustainable modifiers not only improve the engineering performance of asphalt pavements but also support circular economy principles through the utilization of waste-derived and renewable materials. Nevertheless, issues related to compatibility, long-term durability, and large-scale implementation remain important challenges for future applications.

**Table 6:** Sustainable modifiers used in asphalt pavements and their performance relevance

Modifier	Source	Process route	Main benefit	Main limitation	Sustainability relevance
Waste polyethylene (PE)	Plastic packaging, bags, films	Wet process/dry process	Improves stiffness and rutting resistance	Compatibility issues, possible brittleness at low temperature	Reduces plastic waste and supports circular material use
Waste polypropylene (PP)	Consumer plastic products, containers	Wet process/dry process	Enhances high-temperature performance and deformation resistance	Difficult dispersion, variable waste composition	Promotes recycling of thermoplastic waste in road materials
Waste polyethylene terephthalate (PET)	Bottles and packaging waste	Mostly dry process	Increases stiffness and mixture stability	High melting temperature, processing difficulty	Provides a reuse route for non-biodegradable plastic waste
Mixed plastic waste	Municipal and industrial plastic waste streams	Wet process/dry process/hybrid routes	Cost-effective waste utilization and improved mixture performance	Inconsistent composition and reproducibility	Supports large-scale waste diversion from landfills
Crumb rubber	End-of-life tires	Wet process/dry process	Improves elasticity, fatigue life, rutting resistance, and noise reduction	High viscosity, difficult processing, storage instability	Enables valorization of waste tires and reduces environmental burden
Devulcanized rubber	Treated waste tire rubber	Wet process	Better compatibility and interaction with bitumen than untreated crumb rubber	Additional treatment cost and process complexity	Improves waste-rubber reuse efficiency
Natural rubber latex	Renewable rubber source	Wet process	Enhances elasticity and crack resistance	Cost and limited availability in some regions	Renewable alternative to petroleum-based modifiers
Recycled asphalt pavement (RAP) with rejuvenators	Aged asphalt materials	Mixture recycling/binder rejuvenation	Reduces virgin binder demand and material consumption	Quality variation and aging-related stiffness	Highly relevant for resource-efficient pavement recycling
Pyrolysis-derived plastic oils/additives	Thermochemical conversion of waste plastics	Binder modification/rejuvenation	Potential recovery of useful binder-like fractions	Quality control, scale-up, and long-term validation needed	Supports waste valorization and low-carbon material recovery
Bio-based modifiers	Lignin, bio-oils, agricultural by-products	Wet process/hybrid modification	Renewable origin, possible aging resistance improvement	Limited field validation and variability of biomass sources	Supports development of greener and lower-carbon pavements

## 7. Rheological and mechanical properties of polymer-modified bitumen

The rheological and mechanical properties of bitumen are critical parameters governing pavement performance under varying traffic loads and environmental conditions. Since asphalt binders exhibit viscoelastic behavior, their response depends on both temperature and loading frequency. Conventional bitumen often suffers from rutting at high temperatures, fatigue cracking at intermediate temperatures, and thermal cracking at low temperatures. Polymer modification has emerged as one of the most effective approaches for improving the rheological and mechanical performance of asphalt binders by enhancing elasticity, stiffness, deformation recovery, and resistance to aging.^7,18,50,108^

The rheological performance of polymer-modified bitumen strongly depends on both the chemical nature and molecular architecture of the polymer modifier. Ocelić Bulatović *et al.*^58^ demonstrated that elastomeric, plastomeric, and reactive polymers improve bitumen through fundamentally different modification mechanisms. Non-reactive elastomers, particularly SBS, absorb the maltene fraction and swell within the bitumen matrix, forming a continuous three-dimensional physical network that substantially increases the complex modulus, reduces the phase angle, and enhances elastic recovery. Among the investigated elastomers, radial SBS exhibited superior performance because its branched molecular architecture promoted stronger polymer–bitumen interactions and a more stable elastic network than linear SBS. Consequently, SBS-R produced the highest resistance to permanent deformation and the greatest increase in the SHRP critical temperature.

In contrast, EVA acts mainly as a plastomer, increasing stiffness and rutting resistance by forming a rigid dispersed phase rather than an elastic network. Although increasing EVA concentration improves high-temperature performance, its semicrystalline nature leads to melting of crystalline domains at elevated temperatures, disrupting time-temperature superposition and reducing low-temperature flexibility. Therefore, EVA-modified binders generally exhibit higher stiffness but lower elasticity compared with SBS-modified systems.

Reactive polymers such as Elvaloy modify bitumen through a different mechanism by forming covalent bonds between glycidyl methacrylate (GMA) functional groups and polar components of bitumen, particularly asphaltenes. FTIR analysis confirmed the disappearance of epoxy-related absorption bands after modification, providing direct evidence of chemical reactions. This chemical coupling significantly improves storage stability and minimizes phase separation during hot storage. However, excessive reactive polymer content may induce gel formation due to extensive crosslinking, highlighting the need for careful optimization of polymer dosage.

Overall, the study clearly demonstrates that the performance of polymer-modified bitumen is governed not only by polymer concentration but also by the modification mechanism. Elastomeric polymers primarily improve elasticity and fatigue resistance through physical network formation, plastomers mainly enhance stiffness and rutting resistance, while reactive polymers provide superior compatibility and storage stability through chemical bonding. This distinction provides a useful framework for selecting polymer modifiers according to specific pavement performance requirements rather than relying solely on conventional binder properties.

### 7.1 Fundamentals of rheological behavior

Bitumen is a thermorheologically complex material exhibiting characteristics of both viscous liquids and elastic solids. The viscoelastic response is typically described using rheological parameters such as complex shear modulus (G*), phase angle (*δ*), storage modulus (*G*′), and loss modulus (*G*″), which are measured using dynamic shear rheometry (DSR).^7,112^

The complex modulus (G*) represents the overall resistance of the binder to deformation, while the phase angle (*δ*) characterizes the relative contributions of elastic and viscous behavior. Lower phase angles indicate more elastic behavior, whereas higher values correspond to predominantly viscous responses.^7,18^ Polymer modification generally increases G* and decreases *δ*, indicating improved elasticity and greater resistance to permanent deformation under traffic loading.^17,18^

### 7.2 High-temperature performance and rutting resistance

Permanent deformation, commonly known as rutting, is one of the most significant distresses affecting asphalt pavements in warm climates and under heavy traffic. Rutting occurs when asphalt mixtures accumulate unrecoverable strains under repeated loading.^102^

Polymer-modified binders exhibit superior rutting resistance due to the formation of elastic polymer networks that increase stiffness and improve strain recovery. SBS-modified binders, in particular, demonstrate remarkable improvements in high-temperature performance through the formation of a three-dimensional polymer network capable of storing and releasing deformation energy.^50,99^

### 7.3 Fatigue resistance and intermediate-temperature performance

Fatigue cracking results from repeated traffic loading that generates microcracks within asphalt pavements. Over time, these cracks propagate and eventually lead to structural failure.^10^

Polymer modification improves fatigue resistance by increasing binder elasticity and promoting stress redistribution within the pavement structure. SBS, SBR, and crumb rubber modifiers enhance the ability of asphalt binders to absorb cyclic loading energy without crack initiation.^17,24^

The fatigue parameter *G* sin *δ*, introduced in the superpave specification, is commonly used to assess fatigue performance. Lower values of *G* sin *δ* correspond to improved fatigue resistance. Polymer-modified binders generally exhibit reduced fatigue parameters and increased fatigue life compared with conventional binders.^17,51^

The traditional superpave fatigue parameter *G* sin *δ* has important limitations, particularly for polymer-modified binders, because it is determined within the linear viscoelastic region at relatively small strain and does not explicitly account for progressive damage accumulation under repeated loading. Several studies have reported weak correlations between *G* sin *δ* and asphalt-mixture fatigue performance, whereas damage-based approaches such as the Linear Amplitude Sweep (LAS) test combined with viscoelastic continuum damage analysis can provide a more representative assessment of fatigue behavior.^117,118^ Nevertheless, the predictive capability of advanced binder-level fatigue parameters remains dependent on material type, strain level, aging condition, and testing protocol.

Recent studies have shown that nanocomposite PMBs exhibit even greater resistance to fatigue cracking due to enhanced interfacial interactions between the polymer matrix and nanofillers, which improve crack-bridging mechanisms and delay crack propagation.^9,12^

### 7.4 Low-temperature cracking resistance

Thermal cracking occurs when pavement contraction at low temperatures generates stresses exceeding the tensile strength of the asphalt mixture. Conventional binders often become brittle under cold conditions, making them susceptible to crack initiation.^13^ Elastomeric polymers significantly improve low-temperature flexibility by maintaining binder ductility and reducing stiffness. SBS-modified binders exhibit superior stress relaxation behavior and greater resistance to thermal cracking compared with unmodified asphalt.^17^

Low-temperature rheological behavior is typically evaluated using the bending beam rheometer (BBR) and direct tension test (DTT). Polymer-modified binders generally exhibit lower creep stiffness and higher *m*-values, indicating improved stress relaxation capabilities and enhanced resistance to thermal cracking.^54,84^

### 7.5 Elastic recovery and viscoelastic response

One of the most important advantages of polymer modification is the enhancement of elastic recovery. Elastic recovery reflects the ability of a binder to return to its original shape after deformation, thereby reducing the accumulation of permanent strain during service.^18^

SBS-modified binders often demonstrate elastic recovery values exceeding 70%, whereas conventional binders typically exhibit significantly lower recovery capabilities.^18^ The enhanced recovery behavior originates from the formation of interconnected polymer networks and physical crosslinks within the bitumen matrix.^17,58^

Multiple Stress Creep Recovery (MSCR) testing has become a valuable method for evaluating elastic recovery and non-recoverable creep compliance. Polymer-modified binders generally exhibit lower non-recoverable creep compliance (*J*_nr_) values and higher recovery percentages, confirming their superior resistance to rutting and permanent deformation.^19^

### 7.6 Mechanical performance and durability

The improved rheological properties of PMBs can contribute to enhanced mechanical performance at the asphalt-mixture and pavement levels, although the magnitude of this improvement depends on mixture design, aggregate characteristics, aging, loading, and environmental conditions. Increased stiffness at high temperatures, improved flexibility at low temperatures, and superior fatigue resistance collectively contribute to longer pavement service life and reduced maintenance requirements.^48,99^

Crumb rubber-modified binders demonstrate improved energy dissipation and crack resistance due to the swelling of rubber particles and the development of a viscoelastic network.^20^ Similarly, recycled polymer-modified binders can significantly enhance mechanical performance while contributing to sustainability objectives through waste valorization.^23,39,43,109,114^

Nanomodified PMBs have shown exceptional mechanical properties, including increased tensile strength, fracture toughness, and resistance to moisture-induced damage. These improvements result from strong interfacial bonding between nanomaterials, polymers, and bitumen components.^9,12^

Binder-level rheological improvements provide an important mechanistic basis for anticipating asphalt-mixture performance, and several studies have demonstrated meaningful relationships between binder rheological parameters and corresponding mixture responses.^120–123^ In particular, non-recoverable creep compliance (*J*_nr_) obtained from MSCR testing has shown meaningful relationships with asphalt-mixture rutting resistance in laboratory and field evaluations.^120,121^ Similarly, selected binder fatigue parameters obtained from rheological testing have been correlated with mixture fatigue and cracking performance.^122–124^ However, the strength of these relationships depends on the rheological parameter considered, mixture composition, aggregate characteristics, aging condition, loading level, and testing protocol. Therefore, binder-level characterization provides valuable indicators of expected performance, particularly when interpreted together with corresponding mixture-level testing and field observations.

### 7.7 Influence of aging on rheological properties

Oxidative aging causes gradual hardening of asphalt binders due to volatilization and oxidation processes. Aging increases stiffness and brittleness, reducing fatigue resistance and increasing susceptibility to cracking.^22^

Polymer modification can mitigate some of these adverse effects by stabilizing the binder microstructure and reducing oxidation rates. Furthermore, nanomaterials such as graphene and nanoclays can act as diffusion barriers, limiting oxygen penetration and slowing aging processes.^9,23^

Long-term aging studies have demonstrated that PMBs generally retain their rheological properties more effectively than conventional binders, contributing to improved pavement durability over extended service periods.^23^

Experimental studies using FTIR, GPC, rheological analysis, and fluorescence microscopy have demonstrated oxidative degradation of SBS, particularly its polybutadiene segments, accompanied by molecular-weight reduction and progressive deterioration of the polymer network.^96,97^ Consequently, the presence of a polymer does not necessarily ensure improved long-term aging resistance, because the observed response reflects the competing effects of bitumen oxidation and polymer degradation.

Lin *et al.* demonstrated that aging of high-content SBS-modified asphalt involves two competing processes: oxidation and hardening of the bitumen phase and simultaneous degradation of the SBS polymer. Although higher SBS contents reduced carbonyl formation, PAV aging caused a decrease in SBS content and molecular weight, indicating that improved binder aging resistance does not necessarily imply stability of the polymer itself.^96^ More recently, Cao *et al.* confirmed that the polybutadiene segments of SBS are particularly susceptible to oxidative degradation, leading to molecular-weight reduction and progressive destruction of the SBS network; under severe aging, the viscoelastic behavior of the modified binder gradually approached that of aged matrix asphalt.^97^

### 7.8 Role of polymer-containing modifiers in sustainable bitumen production *via* combined oxidation

In recent years, in addition to the use of polymer-containing additives to modify bitumen and improve its characteristics, new technologies have been developed to accelerate the oxidation process, which is one of the ways to obtain high-quality bitumen.

Oxidative and chemical modification of petroleum residues has been investigated as an alternative route for tailoring bitumen properties, including approaches involving petroleum resins and low-molecular-weight reactive compounds. Grynyshyn *et al.* investigated the joint oxidation of petroleum tar with the industrial C9 petroleum resin Piroplast-2 and demonstrated that petroleum resins can participate in bitumen production and modification rather than being introduced exclusively into the finished binder.^125^ The incorporation of petroleum resins increased the softening temperature of the resulting bituminous compositions, although the response depended on the type and properties of the resin, indicating that resin chemistry is an important factor governing modification efficiency.

The importance of resin chemistry was further demonstrated by Bondaletov *et al.*,^126^ who compared aliphatic, cycloaliphatic, and aromatic petroleum resins with their oxidized derivatives as bitumen modifiers. Oxidation of the resins with a hydrogen peroxide/acetic acid system introduced additional functionality and resulted in particularly favorable adhesion of the modified bitumen coatings to metal substrates. The resulting compositions also exhibited low water absorption and high resistance to acidic, alkaline, and saline environments. However, these results were obtained primarily for protective bituminous coatings rather than pavement binders or asphalt mixtures; therefore, their direct implications for pavement rheology and long-term field performance remain to be established.^126^

Chemical modification of the petroleum matrix itself provides another route for controlling the properties of oxidized binders. Gunka *et al.*^127^ investigated the modification of refinery-produced oxidized bitumen with maleic anhydride and identified reaction temperature as the most influential technological parameter. This finding demonstrates that chemical modification cannot be considered independently of processing conditions, because temperature governs the extent and pathway of interactions between the reactive modifier and components of the oxidized bitumen.^127^ Collectively, these studies show that oxidative upgrading is governed not only by oxidation severity but also by feedstock composition, modifier functionality, and reaction conditions. Petroleum resins and reactive organic compounds can therefore influence the chemical and colloidal restructuring of the binder, although improvements demonstrated at the binder or coating level should not automatically be extrapolated to asphalt-mixture and pavement performance without corresponding validation.

Building on these established oxidative and chemical modification approaches, more recent strategies have introduced polymer-containing wastes directly during the oxidation of heavy petroleum residues, combining binder upgrading with waste valorization.

The published research proposed a two-stage combined oxidation of a vacuum residue with crumb rubber: the first stage occurs at 180 °C with a minimal amount of crumb rubber (2%), and the second stage takes place at 260 °C with a substantial amount of crumb rubber (8%). This enabled the acquisition of a modified bitumen with enhanced performance at both high and low temperatures: the rutting parameter values increased from 46 °C to 64 °C, while stiffness dramatically decreased at −40 °C. The primary results indicated that unsaturated and sulfur-containing bonds within the rubber structure led to a significant reduction in oil content and an increase in the presence of condensed aromatic structures. Obtained bitumen binder is characterized by high elasticity and low Fraas point. Prepared asphalt concrete mixture based on the bitumen corresponded to type B according to physical and mechanical parameters. The augmenting the concentration of condensed aromatic structures enhances high-temperature resistance, whereas elastic rubber polymers contribute to low-temperature fracture resistance.^60,128^

Another research report described the oxidative upgrading of heavy oil residues using polymer-containing waste for the sustainable production of bitumen. Oxidation processes were conducted at 250–270 °C with the addition of 2–3 wt% polymer-based waste (polyethylene and polyethylene terephthalate) for 3–4 h, under an air flow of 7 L min^−1^. Contemporary modern spectroscopic investigations demonstrated the emergence of carbonyl and sulfoxide functional groups, signifying the successful oxidative alteration of the bitumen matrix and partial integration of polyethylene fragments. Additionally, confirmed elevated aromaticity and carbonyl content, while also identifying signals generated from polyethylene, indicate compatibility and integration of the polymer waste into the oxidized structure. The thermal and rheological tests indicated that the ideal circumstances for generating high-quality oxidized bitumen required the incorporation of 2% polymer waste at 270 °C for 4 hours. This results in improved physical qualities and chemical stability of bitumen, supporting the feasibility of using polymer-containing waste for bitumen upgrading and providing both environmental and technical advantages.^75^

During combined oxidation, light petroleum fractions can penetrate and swell rubber or polymer phases, facilitating their interaction with the evolving oxidized matrix. Simultaneously, oxidation promotes the formation of oxygen-containing functional groups and increases the proportion of more condensed aromatic structures. Polymer and rubber fragments may undergo chain scission, oxidation, and subsequent recombination or crosslinking, thereby contributing to the restructuring of the binder. However, the relative contribution of these pathways depends strongly on polymer chemistry, oxidation temperature, residence time, and feedstock composition, and therefore requires further mechanistic verification.

### 7.9 Critical comparison and quantitative benchmarking of modification strategies

Direct comparison of polymer-modified bitumen systems is complicated by differences in base-binder grade, modifier concentration, polymer architecture, processing conditions, aging protocol, and rheological test conditions. Nevertheless, quantitative benchmarking of representative systems can reveal important relationships between modification mechanism and engineering response. Table 7 therefore compares selected systems for which sufficiently defined quantitative data are available. The values are intended as representative benchmarks rather than a universal ranking of modifier performance.

**Table 7:** Critical comparison and quantitative benchmarking of representative bitumen modification strategies^a^

Modification strategy	Base binder/modifier conditions	Quantitative benchmark	Mechanistic interpretation	Critical limitation/trade-off	Ref.
Linear SBS (SBS-L)	BIT 70/100; 2–7% SBS-L	SHRP critical temperature increased from 65.1 °C (base binder) to 68.0 °C at 2%, 73.9 °C at 3%, 85.0 °C at 4%, 91.6 °C at 5%, and 100.2 °C at 7%	Swelling in the maltene phase and formation of an elastic physical network increase stiffness and resistance to permanent deformation	Increasing polymer content improves high-temperature response, but processing, compatibility, viscosity, and storage stability must also be considered	58
Radial SBS (SBS-R)	BIT 70/100; SBS-R	At comparable concentrations, SBS-R produced strong increases in critical temperature and elastic response: 67.7 °C at 2%, 75.8 °C at 3%, and 80.6 °C at 4%	Branched/radial architecture promotes a more effective physical network and stronger elastic response	Performance cannot be extrapolated solely from SBS concentration because molecular architecture is also important	58
SBS + EVA + RET	50/70 bitumen; 1% SBS + 1% EVA + 0.5–2.5% RET	Increasing RET decreased penetration and ductility while increasing penetration index, softening point, and elastic recovery; 2.0% RET + 1% SBS + 1% EVA was also examined by FTIR	SBS supplies elasticity, EVA contributes stiffness, while RET introduces reactive coupling with polar bitumen components	Excessive RET concentration increases crosslinking/gelation risk; formulation therefore has a restricted optimum dosage window	67
Latex-modified bitumen emulsion	Latex incorporated into bitumen emulsion	Continuous polymer-rich morphology can be obtained at 2–3 wt% polymer, whereas the conventional hot process generally requires >6 wt% to obtain comparable network continuity	Emulsion breaking promotes efficient polymer-film/network formation at relatively low polymer loading	PMBE production is sensitive to emulsification and drying conditions; excessive drying can alter recovered-binder morphology	85
Polyethylene-waste-assisted oxidative upgrading	Heavy oil residue; 2–3 wt% polymer waste; 250–270 °C; 3–4 h; air flow 7 L min^−1^	Optimum reported at 2 wt% polymer, 270 °C, 4 h; products met OMB 100/130 requirements; thermal stability extended to approximately 350 °C	Unlike conventional physical PMB modification, polyethylene participates in oxidation/aromatization and partial integration into the evolving binder structure	High-temperature/chemical performance is demonstrated, but fatigue, low-temperature cracking, long-term storage, and field performance require further validation	75
Crumb-rubber-assisted combined oxidation	Vacuum residue; 2% CR at 180 °C, followed by 8% CR at 260 °C	Reported rutting-related temperature parameter increased from 46 to 64 °C, accompanied by markedly reduced stiffness at −40 °C	Oxidative restructuring increases condensed aromatic structures, while swollen elastic rubber contributes low-temperature deformability	High modifier content and multistage processing increase technological complexity; broader field validation is required	60 and 128
Petroleum microcoke	BND 100/130; 1 wt% mechanochemically activated petroleum coke	*J*_nr_ 3.2 = 2.08 kPa^−1^; *J*_nr_, diff = 18.54%; binder grade shifted toward BND 70/100	Fine particles/high surface area restrict molecular mobility and reinforce the binder through particle-binder interactions rather than polymer-network formation	Primarily improves high-temperature stiffness/rutting response and does not reproduce the full elastic/fatigue benefits of elastomeric PMBs	66

a Values are representative benchmarks from the cited studies and should not be interpreted as a universal ranking because base-binder composition, modifier dosage, processing conditions, aging state, and test protocols differ among studies.

Table 7 demonstrates that performance enhancement cannot be interpreted independently of the underlying modification mechanism. SBS produces a pronounced improvement in high-temperature performance through formation of an elastic polymer network, and its effectiveness depends not only on concentration but also on molecular architecture. In contrast, reactive systems combine physical reinforcement with chemical coupling, potentially improving compatibility and storage stability but introducing a narrower formulation window because excessive reaction can promote gelation. Latex-modified emulsions illustrate that the processing route itself can alter the concentration required for network formation, with continuous polymer-rich morphology achievable at substantially lower polymer contents than in conventional hot-process PMBs. Polymer-assisted oxidative upgrading represents a further mechanistic distinction because the waste polymer participates in restructuring of the binder during oxidation rather than functioning solely as a dispersed reinforcing phase. Carbonaceous microfillers provide yet another mechanism, predominantly restricting molecular mobility and improving high-temperature resistance without forming an elastomeric network.

These comparisons also help explain apparently conflicting findings in the literature. A modifier that performs favorably in terms of high-temperature stiffness, critical temperature, or *J*_nr_ cannot necessarily be considered advantageous with respect to fatigue or low-temperature cracking resistance. For example, increased stiffness is beneficial for rutting resistance but may be accompanied by reduced low-temperature flexibility, whereas elastomeric network formation can improve recoverable deformation while introducing compatibility and storage-stability challenges. Moreover, differences in base-binder chemistry, polymer molecular architecture and molecular weight, modifier dosage, blending temperature, shear history, aging state, and testing conditions can produce substantially different responses even for nominally similar modifier systems. Accordingly, cross-study comparisons should be interpreted within a processing–structure–property framework rather than on the basis of isolated numerical improvements.

### 7.10 Future perspectives

Future developments in PMB rheology are expected to focus on multifunctional binders capable of simultaneously improving mechanical performance, sustainability, and smart functionality. The integration of nanotechnology, bio-based modifiers, self-healing systems, and machine learning-assisted material design is anticipated to produce next-generation asphalt binders with improved combinations of performance, durability, and sustainability.^9,24^

Advanced rheological characterization techniques, combined with molecular dynamics simulations and artificial intelligence, will further improve understanding of structure–property relationships and facilitate the development of optimized polymer-modified asphalt systems for future transportation infrastructure.

Aitkaliyeva *et al.*^129^ proposed oil sludge as a sustainable rejuvenator for reclaimed asphalt pavement (RAP), demonstrating that petroleum waste can effectively restore the physicochemical properties of aged binders while supporting circular waste management. The addition of 5 wt% oil sludge significantly improved compressive strength, rutting resistance, thermal stability, and binder plasticity, while FTIR and SARA analyses revealed a reduction in oxidation-related carbonyl (C=O) and sulfoxide (S=O) groups, accompanied by a decrease in asphaltenes and an increase in aromatic and resin fractions. SEM observations further confirmed enhanced diffusion and homogenization of the aged asphalt matrix through surface smoothing. These findings indicate that oil sludge functions as a true rejuvenator rather than merely a softening agent by partially restoring the chemical balance of aged bitumen and mitigating oxidative aging, highlighting its potential as an environmentally friendly alternative for high-RAP pavement applications.

## 8. Challenges, gaps, and research needs

Despite remarkable advances in polymer-modified bitumen (PMB) technology over the past several decades, several scientific, technological, economic, and environmental challenges continue to limit its widespread implementation and long-term sustainability. Although PMBs have demonstrated significant improvements in rutting resistance, fatigue life, thermal cracking resistance, and durability compared with conventional binders, many aspects of their behavior remain insufficiently understood. Addressing these challenges is essential for developing next-generation asphalt materials capable of meeting future transportation demands under increasingly complex environmental and operational conditions.

One of the most persistent challenges in PMB technology is the thermodynamic incompatibility between many polymers and bitumen components. Bitumen is a highly heterogeneous colloidal system composed of saturates, aromatics, resins, and asphaltenes, whereas most polymers possess distinct molecular structures and polarity characteristics.^4^ As a result, phase separation frequently occurs during storage, transportation, and prolonged service, particularly at elevated temperatures.

Storage instability remains especially problematic for SBS-, PE-, and PP-modified binders because density differences and limited molecular compatibility promote segregation of polymer-rich and bitumen-rich phases.^5,6^ Although reactive polymers, compatibilizers, and nanomaterials have shown promise in mitigating these issues, the development of universally applicable compatibility enhancement strategies remains an important research priority.^7^

Future studies should focus on understanding molecular-level interactions between bitumen fractions and polymers through advanced characterization techniques and computational modeling. Molecular dynamics simulations and machine-learning-assisted formulation design may provide valuable tools for predicting compatibility and optimizing binder composition before experimental validation.^8^

Aging remains one of the primary factors limiting pavement performance. Oxidative aging, ultraviolet radiation, moisture exposure, and thermal cycling progressively alter binder chemistry, resulting in increased stiffness, reduced flexibility, and greater susceptibility to cracking.^9^

Although polymer modification can improve aging resistance, the long-term evolution of polymer networks under field conditions is not yet fully understood. Many laboratory aging protocols fail to accurately reproduce real environmental conditions, creating uncertainty regarding long-term performance predictions.^10^

Further research is needed to establish reliable accelerated aging methodologies capable of correlating laboratory observations with field performance. Advanced spectroscopic and microscopic techniques should be employed to investigate microstructural evolution during aging and to identify degradation mechanisms affecting different polymer systems.^11^

The growing diversity of polymer modifiers, nanomaterials, recycled materials, and bio-based additives has created significant challenges for standardization. Existing test methods were originally developed for conventional bitumen and may not adequately capture the complex behavior of advanced PMBs.^12^

Future efforts should focus on developing standardized testing protocols capable of evaluating multifunctional binders incorporating self-healing agents, nanomaterials, smart sensing capabilities, and sustainable modifiers. International collaboration among researchers, industry stakeholders, and transportation agencies will be essential for harmonizing these methodologies.

The increasing use of recycled plastics, waste rubber, and bio-based modifiers has substantially improved the sustainability profile of asphalt materials. However, significant uncertainties remain regarding their long-term environmental impacts.^17^

For example, concerns have been raised regarding microplastic generation from plastic-modified pavements, potential leaching of additives, and end-of-life recyclability of modified asphalt materials.^18^ Similarly, while bio-based modifiers reduce dependence on petroleum resources, their environmental benefits depend heavily on feedstock origin, land use, processing methods, and transportation requirements.^19^

Many promising polymer-modification technologies remain confined to laboratory-scale investigations. Transitioning these technologies to industrial-scale production often introduces challenges related to processing complexity, equipment requirements, material consistency, and economic feasibility.

Nanomaterial-enhanced binders illustrate this issue particularly well. Although graphene, carbon nanotubes, nanosilica, and nanoclays have demonstrated outstanding laboratory performance, their large-scale implementation remains constrained by high production costs, dispersion difficulties, and quality-control requirements.

Similarly, bio-based modifiers frequently face challenges associated with feedstock variability and supply chain stability. Future research should prioritize scalable production routes, cost-effective processing technologies, and industrial pilot studies capable of demonstrating practical feasibility.

The emergence of smart pavements incorporating sensors, energy-harvesting systems, self-healing technologies, and intelligent monitoring networks presents exciting opportunities for future transportation infrastructure. However, significant technical barriers remain before widespread implementation can be achieved.

Challenges include sensor durability, power management, data transmission reliability, cybersecurity concerns, and integration with existing infrastructure systems. Furthermore, the interaction between advanced polymers, conductive additives, and sensing components requires further investigation to ensure long-term functionality under traffic loading and environmental exposure.

Future research should explore multifunctional polymer-modified binders capable of simultaneously providing structural performance, sensing capabilities, self-healing functionality, and environmental sustainability.

## 9. Research directions for next-generation PMBs

Based on the knowledge gaps identified in this review, future research should move beyond demonstrating improvements in individual binder properties toward resolving specific mechanistic, validation, and implementation challenges. Priority research tasks include:

(1) Establish quantitative polymer–bitumen interaction criteria by correlating polymer chemistry and molecular architecture with SARA composition, swelling kinetics, interfacial interactions, phase morphology, and storage stability using complementary experimental characterization and molecular simulation.

(2) Develop predictive structure–property models that integrate polymer type, dosage, processing conditions, morphology, and rheological parameters, with machine-learning approaches validated against independent experimental datasets rather than used solely for empirical optimization.

(3) Establish aging protocols representative of field exposure by linking laboratory oxidative, thermal, UV, and moisture aging with changes in polymer molecular structure, binder morphology, rheology, and long-term pavement performance.

(4) Standardize the evaluation of sustainable and multifunctional PMBs, particularly recycled polymers, bio-based modifiers, nanomaterials, self-healing systems, and hybrid binders, for which conventional binder tests may not adequately describe their complex response.

(5) Quantify environmental benefits using performance-normalized life-cycle assessment, incorporating modifier production, processing energy, pavement service-life extension, maintenance requirements, recyclability, and end-of-life scenarios rather than comparing materials only on a mass basis.

(6) Bridge laboratory and field performance through pilot- and full-scale validation, particularly for nanomodified, self-healing, waste-derived, and smart pavement systems. Long-term monitoring should determine whether laboratory improvements in rheology and healing or sensing functionality persist under traffic and environmental exposure.

(7) Establish scale-up and implementation criteria that incorporate processing reproducibility, storage stability, modifier availability, health and environmental considerations, cost, and compatibility with existing asphalt-production infrastructure.

Addressing these tasks would provide a stronger basis for moving next-generation PMBs from laboratory formulation toward predictable, standardized, and practically deployable pavement technologies.

As summarized in Table 8, future asphalt pavements will increasingly integrate advanced materials with digital and intelligent technologies. While self-healing systems, smart sensing, artificial intelligence, and digital twins offer considerable opportunities for improving pavement durability and asset management, their large-scale implementation still requires advances in cost reduction, standardization, and long-term field validation.

**Table 8:** Emerging asphalt technologies for future roads: functions, opportunities, and implementation challenges

Technology	Main function	Expected performance benefit	Key challenge	Development status
Nanomodified asphalt binders	Reinforcement of binder structure and enhancement of thermal, mechanical, and aging properties	Improved rutting resistance, durability, moisture resistance, and thermal stability	Uniform dispersion of nanoparticles, cost, and scale-up complexity	Emerging
Self-healing asphalt	Autonomous or externally stimulated repair of microcracks	Extended service life, reduced maintenance frequency, improved pavement resilience	Healing efficiency under field conditions, material cost, and long-term reliability	Emerging
Smart pavements with embedded sensors	Real-time monitoring of strain, temperature, traffic load, and structural condition	Improved pavement management, early damage detection, and predictive maintenance	Sensor durability, data integration, and installation cost	Developing
Digital pavement inspection systems	Automated detection and assessment of pavement distress using smart materials and digital tools	Faster condition assessment, reduced inspection time, and more accurate maintenance planning	High initial investment, data processing requirements, and standardization	Developing
Polymer-nanomaterial hybrid binders	Combined enhancement of viscoelastic, mechanical, and aging performance	Multifunctional improvement in stiffness, elasticity, durability, and resistance to degradation	Complex formulation design, compatibility, and cost-effectiveness	Emerging
Waste-derived multifunctional binders	Integration of recycled plastics, crumb rubber, and other waste-derived modifiers into binder systems	Sustainable material use, improved performance, and reduced environmental burden	Variability of waste composition, quality control, and reproducibility	Emerging to developing
Self-sensing asphalt materials	Direct sensing of pavement condition through conductive or responsive components	Continuous structural health monitoring and rapid response to damage initiation	Material design complexity, calibration, and signal stability	Early-stage to emerging
Energy-responsive pavements	Pavement systems responsive to external energy sources such as induction or microwave heating	Crack healing, de-icing potential, and enhanced functional performance	Energy efficiency, infrastructure requirements, and field validation	Early-stage to emerging
Low-carbon asphalt technologies	Reduction of environmental impact through bio-based modifiers, recycled inputs, and energy-efficient processes	Lower carbon footprint, resource conservation, and improved sustainability profile	Long-term performance validation and industrial adoption	Developing
Intelligent multifunctional pavement systems	Integration of sensing, healing, durability, and sustainability functions into a single pavement platform	Long-life pavements, lower lifecycle cost, and advanced infrastructure performance	High system complexity, multidisciplinary integration, and commercialization readiness	Early-stage to developing

## 10. Conclusions

Compared with previous reviews that have predominantly addressed polymer-modification mechanisms, individual modifier classes, waste-derived materials, nanomaterials, self-healing asphalt, or smart pavement technologies as separate topics, the present review provides an integrated structure–interaction–performance perspective. Its main novelty lies in linking modifier chemistry and processing conditions with polymer-bitumen interactions, swelling and phase morphology, network formation, rheological response, storage stability, and ultimately mixture- and pavement-level performance. A further distinctive aspect is the inclusion of polymer-assisted oxidative upgrading as an alternative modification pathway, in which waste polymers can participate in the chemical and colloidal restructuring of the binder during oxidation rather than functioning solely as dispersed reinforcing phases. The review also connects conventional PMB science with sustainable and multifunctional approaches, including recycled and bio-based modifiers, nanomaterials, self-healing systems, and smart pavement technologies, while critically considering their technological maturity, field validation, and implementation limitations.

This review demonstrates that the performance of polymer-modified bitumen cannot be attributed to polymer type or dosage alone but results from a coupled relationship among modifier chemistry, processing conditions, polymer–bitumen interactions, phase morphology, and the resulting rheological response. Elastomers, plastomers, reactive polymers, recycled materials, and hybrid modifiers operate through distinct mechanisms, ranging from swelling and physical network formation to stiffening, chemical coupling, and particle reinforcement. These mechanistic differences explain why similar improvements in individual binder properties do not necessarily translate into equivalent fatigue, low-temperature, storage-stability, or pavement-level performance.

The reviewed evidence further shows that sustainability should be evaluated together with engineering durability rather than solely through the replacement of virgin materials with recycled or renewable alternatives. Waste-derived polymers, crumb rubber, bio-based modifiers, polymer-assisted oxidative upgrading, and nanomaterials provide important opportunities, but their benefits remain dependent on compatibility, processing reproducibility, aging resistance, service-life extension, and environmental performance. Similarly, self-healing and smart pavement technologies broaden the functionality of modified asphalt, although their technological maturity and level of field validation vary considerably.

Overall, the structure–interaction–performance framework developed in this review emphasizes that next-generation PMB design requires integration of molecular and microstructural understanding with rheological characterization, mixture-level validation, durability assessment, and life-cycle considerations. Such an integrated approach provides a more reliable basis for translating advances in modifier chemistry into durable and sustainable pavement systems.

## Author contributions

Yernar Kanzharkan: conceptualization, methodology, investigation, data curation, writing – original draft, writing – review & editing, visualization. Yerbol Tileuberdi: supervision review & editing. Yerdos Ongarbayev: supervision review & editing, Yerzhan Imanbayev: project administration, funding acquisition. Elmira Nurgaziyeva: conceptualization, methodology, formal analysis, investigation, validation, resources, writing – original draft – review & editing. Munziya Abutalip: conceptualization, methodology, formal analysis, investigation, validation, resources, writing – original draft – review & editing. All authors have read and agreed to the published version of the manuscript.

## Conflicts of interest

The authors declare no conflicts of interest.

## Data Availability

No primary research results, software, or code have been included, and no new data were generated or analysed as part of this review.
